# Genetic association and machine learning improve the prediction of type 1 diabetes risk

**DOI:** 10.1038/s41588-026-02578-y

**Published:** 2026-04-30

**Authors:** Carolyn McGrail, Timothy J. Sears, Emily N. Griffin, Alexandra L. Ghaben, Patrick Smadbeck, Jason Flannick, Parul Kudtarkar, Hannah Carter, Kyle Gaulton

**Affiliations:** 1 Biomedical Sciences Graduate Program, University of California, San Diego, La Jolla, CA USA; 2 Bioinformatics and Systems Biology Program, University of California, San Diego, La Jolla, CA USA; 3 Department of Pediatrics, University of California, San Diego, La Jolla, CA USA; 4 Programs in Metabolism and Medical and Population Genetics, Broad Institute of MIT and Harvard, Cambridge, MA USA; 5 Division of Genetics and Genomics, Boston Children’s Hospital, Boston, MA USA; 6 Department of Pediatrics, Harvard Medical School, Boston, MA USA; 7 Moores Cancer Center, University of California, San Diego, La Jolla, CA USA; 8 Department of Medicine, University of California, San Diego, La Jolla, CA USA; 9 Pediatric Diabetes Research Center, University of California, San Diego, La Jolla, CA USA

**Keywords:** Type 1 diabetes, Genome-wide association studies, Genetics research

## Abstract

Type 1 diabetes (T1D) has a large genetic component, and expanded genetic studies of T1D can enhance biological and therapeutic discovery and improve risk prediction. Here we performed genome-wide genetic association and fine-mapping analyses in 20,355 T1D and 797,363 nondiabetic individuals of European ancestry and in 10,107 T1D and 19,639 nondiabetic individuals at the MHC locus, which identified 160 risk signals. We trained a machine learning model, T1GRS, to predict T1D using genetic risk, which improved classification in Europeans and performed similarly in African Americans, compared to previous scores. T1GRS particularly improved prediction in T1D, with fewer high-risk HLA haplotypes and more complex risk profiles, and revealed 154 nonlinear interactions between MHC and non-MHC loci. Finally, we identified four genetic subclusters based on T1GRS features with significant differences in age of onset and diabetic complications. Overall, improved genetic discovery and prediction will have wide clinical, therapeutic and research applications for T1D.

## Abstract (web summary)

Genome-wide association and fine-mapping analyses of type 1 diabetes (T1D) identify multiple genetic risk signals. Furthermore, a machine learning model, T1GRS, improves the prediction of T1D in individuals with complex risk profiles and identifies genetic subgroups.

## Main

Type 1 diabetes (T1D) is an autoimmune disease characterized by destruction of pancreatic β cells, resulting in lifelong dependence on insulin therapy^1^. The natural history of T1D suggests that the disease occurs in genetically susceptible individuals exposed to environmental triggers, leading to the development of islet-specific autoantibodies and autoreactive T cells and progressive loss of insulin secretory function, although the underlying etiology is not fully understood^1^. The timing of T1D onset is variable, typically occurring during early life or adolescence, but also commonly seen in adults^2^. Islet-specific autoantibodies are used as molecular biomarkers of autoimmunity and can be detected in the blood before the onset of clinical T1D^3^. However, islet autoantibodies may be transient, are less frequently found in adult-onset cases and are not fully predictive of T1D^3,4^. With the adoption of disease-modifying therapies such as teplizumab^5,6^, identifying susceptible individuals and improving risk prediction is of increasing clinical interest and utility.

There is a large heritable component to T1D and genetic risk factors can identify individuals with increased susceptibility to T1D. Genetic variants in class I and II Major Histocompatibility Complex (MHC) genes are the largest T1D risk factors^7,8^, most notably HLA-DRB1*03:01 ~ HLA-DQB1*02:01 (DR3-DQ2) and HLA-DRB1*04:01 *~* HLA-DQB1*03:02 (DR4-DQ8) haplotypes, which, when inherited together, increase risk of T1D over 16-fold^7^. Beyond the MHC locus, T1D is highly polygenic, with over 90 additional risk loci identified, including *INS* and *PTPN22* (refs. ^9–13^). The heritability of T1D, however, remains incompletely described^14^, implying additional T1D loci remain to be discovered. In addition, risk loci often contain many associated variants due to linkage disequilibrium (LD), which indicates the presence of one or more unknown causal variants^15^. Larger genetic association studies can boost statistical power to detect new risk loci and fine-mapping analyses can narrow candidate causal variants within these loci. Together, these approaches can advance our understanding of the genetic basis of T1D^15^.

Genetic variants associated with T1D can be used to construct genetic risk scores (GRS)^16,17^, which summarize an individual’s genetic risk and can predict the development of T1D. The utility of a GRS as a diagnostic tool may, for example, aid in the selection of individuals for preventive therapies, complement the interpretation of blood biomarkers and distinguish between forms of diabetes^16–20^. Previous studies have developed risk scores for T1D that accurately discriminate T1D from nondiabetes and from type 2 diabetes (T2D)^16,17^. Improvements to existing T1D GRSs, however, may further enhance their utility and accessibility. Current T1D GRSs are typically calculated as the additive sum of individual risk allele effects and, outside of known interactions among class II MHC alleles, generally do not consider nonlinear interactions between variants, particularly at other loci genome-wide^16,17,21^. Furthermore, previous T1D GRSs are not straightforward to calculate using genotypes imputed from modern reference panels and require phasing of HLA haplotypes and use of proxy variants^16,17^, which may limit their utility.

**Fig. 1:**
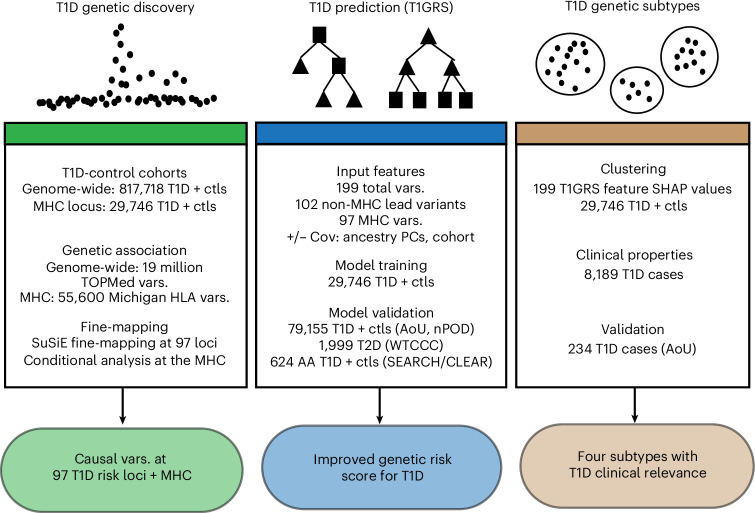
Overview of study design. We first performed a genome-wide association and fine-mapping study of T1D, which together revealed candidate causal variants for 160 signals at 97 risk loci plus the Major Histocompatibility Complex (MHC) locus. We next used risk variants genome-wide and at the MHC locus to train a machine learning model (named T1GRS) to predict T1D, which we validated using multiple independent cohorts of T1D, T2D and nondiabetic individuals from both European and African American ancestry. Finally, we performed clustering of individuals based on features from T1GRS, which revealed four genetic subtypes with distinct clinical properties that we validated in an independent cohort. AA, African American; ctls, controls; Cov, covariates; PCs, principal components; vars., variants.

In this study, we performed genetic discovery, prediction and subgrouping of T1D (Fig. 1). First, we performed a genetic association and fine-mapping study of T1D, which revealed 160 T1D risk signals. We then used variants at these signals to train a machine learning model, T1GRS, to predict T1D. The T1GRS model exceeded predictive and diagnostic ability compared to T1D GRS2 (ref. ^16^), particularly in individuals without high-risk HLA haplotypes and with more complex risk and revealed significant interactions between risk variants. Finally, we identified genetic subgroups of T1D based on T1GRS features, which exhibited distinct clinical profiles, including variations in age at onset and disease-related complications.

## Results

### Genome-wide association and fine-mapping identify new T1D signals

**Fig. 2:**
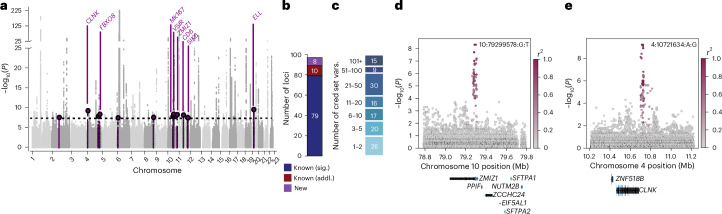
Genome-wide association and fine-mapping of T1D. **a**, Genome-wide association analysis of T1D in *n* = 817,718 (T1D, *n* = 20,355; nondiabetic, *n* = 797,363) individuals. *P* values were from a meta-analysis of T1D association statistics from two-sided Firth bias-corrected LogReg. New loci with uncorrected *P* < 1 × 10^−8^ in the discovery or replication data are colored purple and labeled, while putative loci with uncorrected *P* < 5 × 10^−8^ are colored purple but not labeled further. Variants with a *P* < 1 × 10^−225^ were capped at this threshold for display. **b**, In total, there were 97 T1D loci, including 89 previously established loci (79 loci reaching significance in this study and 10 additional loci) and 8 new loci. **c**, Number of credible set variants for the 133 signals identified after fine-mapping 97 T1D loci with SuSiE. **d**,**e**, Locus plots of *P* values from the same T1D association analysis described in **a** at the new loci *ZMIZ1* (**d**) and *CLNK* (**e**). The color of each variant represents the linkage disequilibrium (*r*^2^) with the lead variant at the locus. Sig.,significant; addl., additional; cred, credible.

We performed a T1D association study involving 817,718 individuals of European ancestry (T1D, *n* = 20,355; nondiabetics, *n* = 797,363) using a meta-analysis of nine cohorts (Supplementary Table 1). Variants were imputed into the TOPMed v3 reference panel (for UK Biobank, the HRC reference panel) and up to 19 million variants with minor allele frequency (MAF > 0.1%) were considered in association tests. In total, we identified variants at 79 known loci and 8 previously unreported loci at a genome-wide threshold of *P* < 1 × 10^−8^ (Supplementary Table 2 and Fig. 2a).

We performed fine-mapping at 97 T1D loci, including the 79 known and 8 new loci, as well as 10 previously known T1D loci not significant in this study (Fig. 2b and Supplementary Table 2). Across these 97 loci, we identified 133 independent signals, where several loci, such as *IL2RA*, *INS*, *PTPN22* and *IFIH1*, exhibited more than 2 signals (Supplementary Table 3). At each signal, we derived ‘credible sets’ of likely causal variants (Supplementary Table 3). Over half (75/134) of credible sets had 15 or fewer variants and over one-third (46/134) had 5 or fewer variants (Fig. 2c). Overall, there was a high concordance with previous fine-mapping, where ~97% of signals at known loci overlapped those in a previous study^9^. At several signals, fine-mapping resolution was improved. For example, at the *CTRB2* locus, fine-mapping resolved a single variant (rs55993634; posterior inclusion probability (PIP) = 0.989), whereas this same variant previously was part of a larger credible set^9^. At a few loci, there was limited overlap between credible sets and those in previous studies, notably at the *UBASH3A* locus, where reported signals are in partial LD^9^.

We annotated the credible set variants at the eight newly identified loci to understand their potential molecular mechanisms. At three loci, a candidate variant mapped within a protein-coding gene and the remaining five mapped to intergenic sequences (Supplementary Table 4). At the *ZMIZ1* locus, candidate variants mapped in the intron of *ZMIZ1*, which is also involved in T2D risk and its product affects β-cell function and glucose homeostasis^22^ (Fig. 2d). Loci that were fully intergenic included variants near *CLNK* (Fig. 2e), which encodes a mast cell signal transducer that contributes to the regulation of immunoreceptor signaling. Credible set variants at this locus overlapped accessible chromatin sites in both pancreatic and immune cells, suggesting they alter gene regulation.

In total, these results provide expanded genetic association and fine-mapping of T1D and revealed new risk loci and independent signals.

### Fine-mapping of the MHC locus identifies additional risk signals

Given the importance of the MHC locus to T1D risk, we performed detailed fine-mapping to identify additional T1D signals at this locus. We used genotypes from 29,746 individuals of European ancestry (T1D, *n* = 10,107; nondiabetic, *n* = 19,639) from five cohorts available for HLA imputation (Supplementary Table 1;Methods). After quality control, we imputed variants into the Michigan HLA reference panel containing 55,614 variants, including HLA alleles at the first-field and second-field levels, amino acid residues and intergenic variants (Methods) and considered variants with MAF > 1% in analyses.

**Fig. 3:**
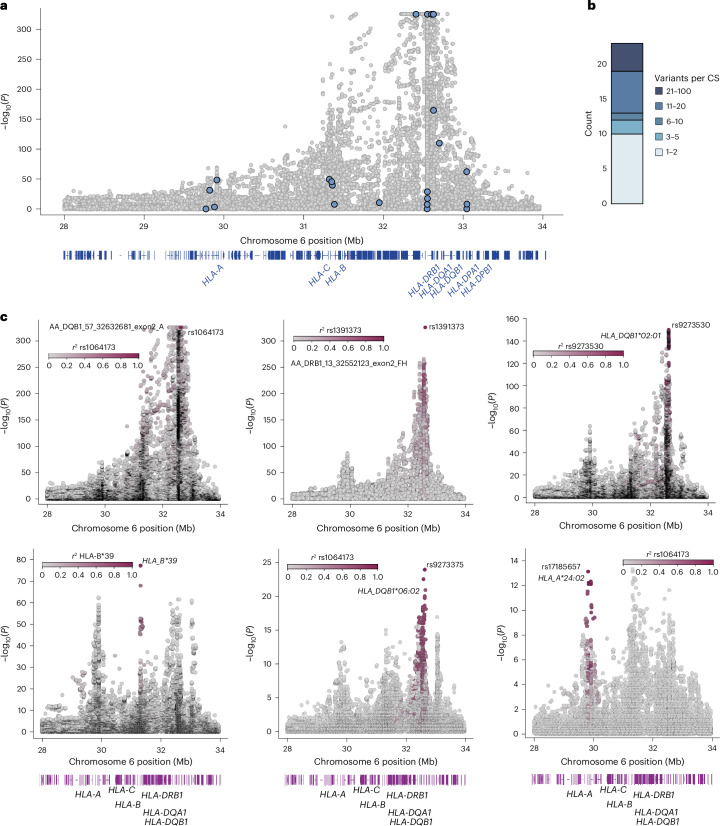
Genetic association of T1D at the MHC locus. **a**, T1D association analysis of variants at the MHC locus using two-sided Firth bias-corrected LogReg. *P* values are from the marginal T1D association statistics from meta-analysis of *n* = 29,746 (T1D, *n* = 10,107; nondiabetic, *n* = 19,639) individuals. The lead variant for each signal is colored blue and signals with *P* < 1 × 10^−325^ were capped at this threshold for display. The gene map shows the location of class I and II HLA genes. **b**, The number of variants per credible set is shown for the 23 signals identified in conditional fine-mapping. **c**, Locus plots showing variant *P* values for conditional association signals at the MHC locus from two-sided Firth bias-corrected LogReg of *n* = 29,746 individuals. Locus plots are colored by linkage (*r*^2^) to the lead variant for each signal. *HLA-DQB1:A57* refers to variants in *HLA-DQB1* at position 57 and *HLA-DRB1:FH13* refers to variants in *HLA-DRB1* at amino acid position 13. The gene maps show the locations of class I and II HLA genes. CS, credible set.

Due to the extreme associations and extensive LD, methods such as SuSiE are less reliable when fine-mapping the MHC locus. We therefore performed a stepwise conditional analysis by iteratively including the most significant variant as an additional covariate and reperforming association tests until no variants were significant (Methods). This revealed 23 independent signals associated with T1D at *P* < 5 × 10^−8^ (Fig. 3a,b and Supplementary Tables 5 and 6). For each signal, we derived ‘credible sets’ of likely causal variants using a Bayesian approach (Methods). As expected, signals primarily consisted of variants in LD with known class I and class II MHC T1D risk alleles^7,23,24^ (Fig. 3c and Supplementary Table 5). We also identified signals not linked to known T1D risk alleles, including a noncoding signal (rs9276235) between *HLA-DQB1* and *HLA-DQA2* (Supplementary Fig. 1a).

As new signals at the MHC locus may be obscured by partial LD with known risk variants, we performed a second round of conditional analyses at the HLA locus after preconditioning on 70 known class I and class II MHC risk alleles and 20 known DR3-DQ2 and DR4-DQ8 haplotypic and allelic interactions (Methods; Supplementary Tables 7–9). These analyses revealed four additional signals (*P* < 5 × 10^−8^) not linked to any known HLA risk alleles (Supplementary Fig. 1b), including amino acid residue 71 in *HLA-DRB1* (ref. ^25^). The other three signals were noncoding and we annotated credible sets for these signals using accessible chromatin sites and transcription factor (TF) binding motifs. For example, at a new signal near *HLA-A*, variant rs7763052 (PIP = 0.173) overlapped an accessible chromatin site in effector CD8^+^ T cells and NK cells (Supplementary Fig. 1c)^26^. At another new signal located upstream of *HLA-DRB1*, variant rs9270965 (PIP = 0.987) altered the binding of the TF SOX3 (ref. ^27^).

Altogether, fine-mapping resolved independent signals at the HLA locus, including both coding and noncoding signals that had not previously been implicated in T1D.

### Machine learning model improves the classification of T1D

We next developed a machine learning model based on gradient boosting to predict T1D using risk alleles at the MHC locus and genome-wide, which we named T1GRS. A total of 199 variants were considered in T1GRS, including lead variants at 102 non-MHC loci (including 5 putative loci), 70 known HLA variants and 27 HLA variants identified in conditional analysis (Methods; Supplementary Table 10). We trained two versions of T1GRS using (1) 199 variants in addition to sex, four genotyping PCs and cohort label (termed ‘T1GRS-cov’) and (2) only the 199 variants, for settings where the other variables are not available or relevant (termed ‘T1GRS-var’). For each of these T1GRS models, we further trained a separate ‘MHC-only’ model using the 97 HLA variants and a ‘non-MHC’ model using the 102 variants outside the HLA locus.

**Fig. 4:**
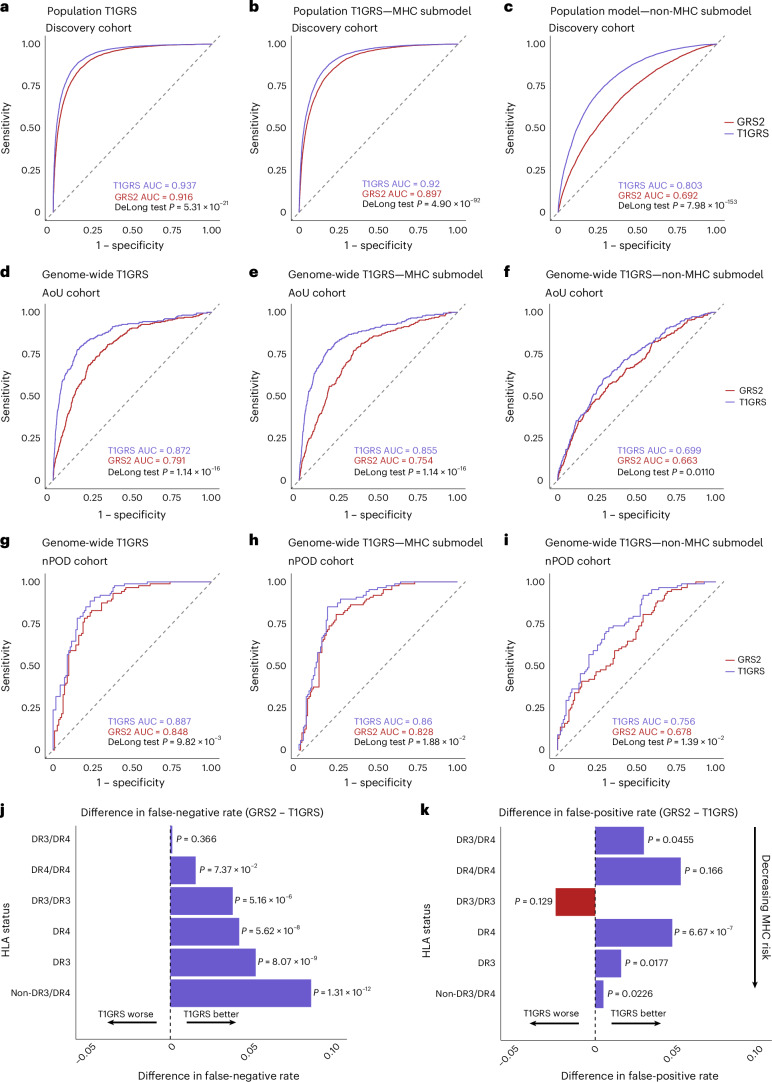
Prediction of T1D using machine learning of variant genotypes. Receiver operating characteristic curves assessing the accuracy of predicting T1D from unaffected individuals using T1GRS and GRS2. The AUC for T1GRS is colored purple, while the existing GRS2 is colored red. **a**–**c**, The AUCs for T1GRS-cov and GRS2 are shown in the discovery dataset for all variants (**a**), MHC-only variants (**b**) and non-MHC genome-wide variants (**c**). **d**–**f**, Validation using T1GRS-var was performed in the NIH AoU Research Cohort for all variants (**d**), MHC-only variants (**e**) and non-MHC genome-wide variants (**f**). **g**–**i**, Validation in the nPOD cohort comparing T1GRS-var to GRS2 for all variants (**g**), MHC-only variants (**h**) and non-MHC genome-wide variants (**i**). *P* values comparing the predictive ability of GRSs for **a**–**i** were calculated using a two-sided DeLong test. **j**, The difference in false-negative rates (GRS2 − T1GRS-cov) for the discovery cohorts. *P* values were calculated by a two-sided McNemar’s test for each category. **k**, The difference in false-positive rates (GRS2 − T1GRS-cov) for the discovery dataset. *P* values were calculated by a two-sided McNemar’s test for each category.

We evaluated the ability of T1GRS to predict T1D in 29,746 individuals of European ancestry (T1D, *n* = 10,107; nondiabetic, *n* = 19,639) from our discovery cohorts using tenfold cross-validation (Methods; Supplementary Table 1). For comparison, we also calculated GRS2 scores in the same individuals. The T1GRS-cov model had strong discrimination of T1D, with an area under curve (AUC) of 0.937 and average precision (AP) of 0.879 (Fig. 4a, Supplementary Fig. 2a and Supplementary Table 11), with similar performance using MHC-only variants (AUC = 0.920, AP = 0.847; Fig. 4b and Supplementary Fig. 2b) and reasonable discrimination using non-MHC variants although with reduced average precision (AUC = 0.803, AP = 0.662; Fig. 4c and Supplementary Fig. 2c). Compared to GRS2 (ref. ^16^), all three T1GRS-cov models showed significant improvements in T1D prediction, most notably the non-MHC model (T1GRS, AUC = 0.803; GRS2, AUC = 0.692; *P* < 0.0001; Fig. 4a–c and Supplementary Fig. 2a–c). The T1GRS-var model also had significantly improved performance (all *P* < 0.0001) relative to GRS2 using all variants (T1GRS, AUC = 0.923; GRS2, AUC = 0.916), MHC-only variants (T1GRS, AUC = 0.903; GRS2, AUC = 0.897) and non-MHC variants (T1GRS, AUC = 0.718; GRS2, AUC = 0.692; Supplementary Fig. 3a–c and Supplementary Table 11). We determined the potential of T1GRS as a diagnostic and the maximum Youden index for T1GRS was 0.733 at a score of 0.574 (89% sensitivity, 84% specificity for T1D), which is an improvement over GRS2 (ref. ^16^) (Table 1 and Supplementary Table 12) and T1GRS scores had cleaner separation between T1D and nondiabetes (Supplementary Fig. 3d).

**Table 1:** T1D prediction using T1GRS

T1GRS probability	T1D centile	Nondisease centile	Sensitivity (%)	Specificity (%)	Youden index
0.3296	5	73.06	95.01	73.05	0.6806
0.5355	10	82.78	90.01	82.78	0.7279
0.6641	15	87.8	85.01	87.8	0.7281
0.7412	20	90.75	80	90.75	0.7075
0.8007	25	92.95	75	92.96	0.6796
0.8449	30	94.53	70	94.53	0.6453
0.8751	35	95.74	65.01	95.73	0.6074
0.8983	40	96.53	60.01	96.53	0.5654
0.9177	45	97.34	55.01	97.34	0.5234
0.9322	50	97.94	50.01	97.93	0.4794
0.9729	75	99.48	25.01	99.47	0.2448
0.9912	95	99.95	5	99.95	0.0495

Sensitivity, specificity and Youden index for T1D at different T1GRS probability thresholds.

As training cohorts may not reflect the true ability of the T1GRS model to predict T1D, we next evaluated T1GRS using independent cohorts. We used cohorts available from the NIH All of Us (AoU) and the Network for Pancreatic Organ Donors with Diabetes (nPOD), defining T1D and nondiabetic individuals of European ancestry (Methods). The performance of the T1GRS-var model was overall reduced compared to the discovery cohorts (AoU, AUC = 0.872; nPOD, AUC = 0.887; Fig. 4d,g), with similar patterns in the MHC (AoU, AUC = 0.855; nPOD, AUC = 0.860; Fig. 4e,h) and the non-MHC models (AoU, AUC = 0.699; nPOD, AUC = 0.756; Fig. 4f,i). However, the T1GRS model significantly outperformed GRS2 across all comparisons, with the most pronounced difference in AoU (Fig. 4d; T1GRS, AUC = 0.872; GRS2, AUC = 0.791; *P* < 0.0001). These results demonstrate that T1GRS significantly improves the prediction of T1D in nondiabetic individuals in independent cohorts.

GRS has previously shown the ability to distinguish T1D from T2D, which can reduce misdiagnosis^17^. We tested the ability of T1GRS-var to classify T1D and T2D using 1,999 T2D individuals from Wellcome Trust Case Control Consortium (WTCCC1) and T1D individuals from nPOD (Methods) and T1GRS accurately differentiated T1D and T2D (Supplementary Fig. 3e–g). Finally, although T1GRS was trained on variants identified in European ancestry individuals, we evaluated its ability to predict T1D in African American individuals (T1D, *n* = 220; nondiabetic, *n* = 404). The performance of T1GRS was identical to a published score derived from an African American T1D GWAS^28^ (T1GRS, AUC = 0.845; African American, AUC = 0.846; *P* = 0.961; Supplementary Fig. 3h,i).

Overall, T1GRS can predict T1D from nondiabetes and T2D at rates beyond current standards in Europeans and T1D at current standards in African Americans.

### Improved performance of T1GRS across MHC and non-MHC variants

We next investigated the T1GRS performance to identify areas for improvement in T1D prediction compared to previous methods.

First, we determined the performance of T1GRS in individuals across different combinations of HLA-DR3/DR4 haplotypes. Overall, there was a significantly lower rate of false-negative T1D classifications in T1GRS relative to GRS2 with a decreasing number of DR3/DR4 haplotypes (Fig. 4j). Similarly, the false-positive rate was also generally lower in T1GRS across DR3/DR4 combinations (Fig. 4k). The greatest area of improvement for T1GRS relative to GRS2 was among individuals without HLA-DR3/DR4 haplotypes. In these individuals, T1GRS significantly outperformed GRS2 (*P* < 0.0001) using all variants (AUC = 0.786 versus 0.764), MHC-only variants (AUC = 0.738 versus 0.706) and non-MHC variants (AUC = 0.736 versus 0.713; Supplementary Fig. 3j–l). In addition, imputing MHC alleles directly increased the proportion of T1D individuals with high-risk HLA-DR3/DR4 haplotypes correctly predicted by T1GRS compared to proxy-based approaches^16^ (Supplementary Fig. 3m).

We next investigated the effects of the additional genome-wide loci included in T1GRS on T1D prediction. Several newly added loci contain rare variants with larger effects on T1D, such as *CEL* (odds ratio (OR) = 2.8, MAF = 0.0015). There was significantly elevated average percentile of non-MHC T1D individuals with at least one copy of the *CEL* risk allele in T1GRS relative to their average percentiles in GRS2 (paired *t* test, *P* < 0.0001; Supplementary Fig. 4a). More broadly, the average non-MHC percentile across all T1D individuals was significantly higher in T1GRS compared to GRS2 (59.63% versus 49.98%; paired *t* test, *P* < 0.0001). Furthermore, individuals above the 50th percentile for T1GRS but below the GRS2 50th T1D percentile (Supplementary Fig. 4b) had higher non-MHC scores in T1GRS, highlighting the role of genome-wide risk in improving T1D prediction in T1GRS.

These results demonstrate that T1GRS improves prediction in individuals without high-risk HLA haplotypes and carrying additional risk genome-wide.

### Prediction of T1D using T1GRS identifies nonlinear interactions

A key benefit of machine learning models over additive scores is their improved ability to identify nonlinear interactions among features. To highlight the value of interactions for T1D prediction, we compared T1GRS with a logistic regression (LogReg) model using the same 199 variants and covariates (Supplementary Fig. 4c–e). Compared with LogReg, T1GRS significantly improved (*P* < 0.0001) classification of T1D (T1GRS, AUC = 0.937; LogReg, AUC = 0.896), as well as using MHC-only (T1GRS, AUC = 0.92; LogReg, AUC = 0.876) and non-MHC variants (T1GRS, AUC = 0.803; LogReg, AUC = 0.725).

**Fig. 5:**
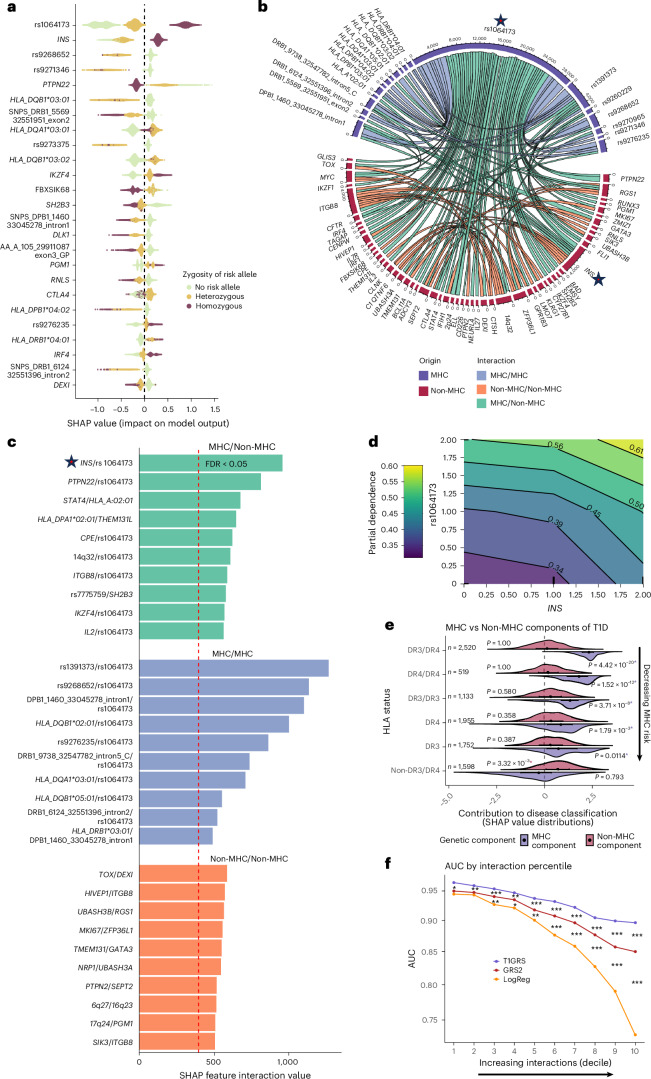
T1GRS reveals nonlinear interactions and genetic complexity in T1D. **a**, SHAP analysis with feature importance in the top 25 features for discovery cohort individuals assessed with T1GRS-cov. Colors indicate the contribution of 2, 1 or 0 copies of the risk allele. Positions at either end of the *x* axis indicate the greatest impact on T1D classification. **b**, Chord diagram maps the strongest interactions between T1GRS variants. Three categories are highlighted—blue represents MHC–MHC interactions, green represents MHC–non-MHC interactions and orange indicates non-MHC–non-MHC interactions. Variants with a key interaction, rs1064173/*INS*, are highlighted with a star. **c**, Top ten feature interactions ranked by interaction value shown for each previously defined category. FDR < 0.05 threshold is indicated by a dashed red line. The same key interaction, rs1064173/*INS*, is highlighted with a star. **d**, Two-dimensional partial dependence plot of *INS* and rs1064173 as a function of allele count. Allele counts between 0 and 1 and 1 and 2 have been interpolated for visualization purposes. **e**, Dueling density plots of SHAP values, averaged across individuals for each feature for MHC and non-MHC variants, where individuals are binned by HLA-DR3 or HLA-DR4 haplotype status. A blue asterisk indicates that the MHC distribution is significantly different from 0, while a red asterisk indicates a significant difference for the non-MHC distribution. *P* values were calculated by a one-sample, two-tailed *t* test. Error bars on the plot represent the interquartile range. **f**, AUC values for T1GRS (purple), GRS2 (red) and a LogReg (orange) are plotted at increasing Complexity Score deciles (Methods). Two-sided DeLong tests—*P < 0.05, **P < 0.001, ***P < 0.0001—for T1GRS versus GRS2 and GRS2 versus LogReg, arranged between AUC points at each decile. Specific *P* values for AUC and DeLong tests can be found in Supplementary Table 14.

To determine the relative importance of specific features in T1GRS to T1D prediction, we used Shapley Additive Explanations (SHAP) analysis (Methods). The most important features for T1GRS predictions included rs1064173, which tags *HLA-DQB1* alleles lacking an aspartic acid at position 57, as well as lead variants at the *INS* and *PTPN22* loci (Fig. 5a and Supplementary Fig.  5a,b). We noted extensive variability in SHAP values for many risk variants across individuals (Fig. 5a), indicating the presence of interactions with other variants. To identify variant interactions, we calculated Shapley interaction (SHAP int) values, which quantify the joint contribution of a pair of variants to model predictions. For each interaction, we calculated *z* scores from the SHAP int values by comparing with a background distribution, followed by false discovery rate (FDR) correction (Fig. 5b,c). In total, 154 variant pairs had significant interactions (FDR < 0.05; Methods; Fig. 5b,c and Supplementary Table 13). The strongest interaction was between amino acid 57 of *HLA-DQB1* (tagged by rs1064173) and amino acid 13 of *HLA-DRB1* (tagged by rs1391373; SHAP int *z* score = 12.9), which reflects the known interaction of HLA-DR3 and HLA-DR4 haplotypes (Fig. 5b,c)^25^. Position 57 of *HLA-DQB1* interacted with other HLA alleles, including HLA-DQB1*02:01 (*z* score = 10.0), the protective HLA-DRB1*15:01 ~ HLA-DQB1*06:02 haplotype (tagged by rs9268652; *z* score = 11.5) and the noncoding signal rs9276235 located between *HLA-DRB1* and *HLA-DQA2* (*z* score = 8.4; Fig. 5b,c).

We also identified significant interactions involving T1D risk variants at the MHC locus and other genome-wide risk loci. For example, there was a significant interaction between the *INS* locus and amino acid 57 of *HLA-DQB1* (Fig. 5c; *z* score = 9.6). While the marginal effects of each feature individually on T1D risk were approximately additive (Supplementary Fig. 5c,d), there was a clear change in the allelic effects of one variant dependent on the other (Fig. 5d). We also identified significant interactions (FDR < 0.10) between different pairs of non-MHC loci (Fig. 5c). Overall, many top interactions appeared multiplicative, although there were also potential dominant or recessive interactions (Supplementary Fig. 6).

The presence of HLA-DR3 and/or HLA-DR4 haplotypes is strongly associated with the risk of developing T1D, and we next sought to determine the drivers of T1D risk in individuals with different combinations of HLA-DR3/DR4 haplotypes. We derived the overall importance of MHC and non-MHC variant components to T1D predictions for individuals with each DR3/DR4 combination using the cumulative SHAP values of each component (Fig. 5e). The importance of MHC variants to T1D prediction was highest in HLA-DR3/DR4 heterozygotes, as expected, with decreasing importance across homozygotes for DR3 or DR4, one DR3 or DR4 haplotype and no DR or DR4 haplotype. Conversely, the importance of non-MHC variants was significantly elevated in non-DR3/DR4, supporting the increased importance of non-MHC loci genome-wide to T1D risk in individuals without high-risk HLA alleles.

We further examined how the number of interactions within an individual affects T1D prediction. We defined a ‘complexity’ metric for all individuals as the total displacement of SHAP values (Methods). We ranked individuals by this complexity metric and calculated T1D predictions using T1GRS, GRS2 and a LogReg model across deciles of complexity. Although T1GRS significantly outperformed GRS2 across all deciles, the performance difference was pronounced with increasing complexity (Fig. 5f and Supplementary Table 14). LogReg showed an even larger decrease in performance among individuals with increasing complexity than among those with T1GRS or GRS2, further emphasizing the importance of interactions in predicting T1D.

Overall, these results reveal nonlinear interactions between MHC and non-MHC loci and highlight their importance in improving the prediction of T1D.

### Clustering by genetic features reveals T1D heterogeneity

**Fig. 6:**
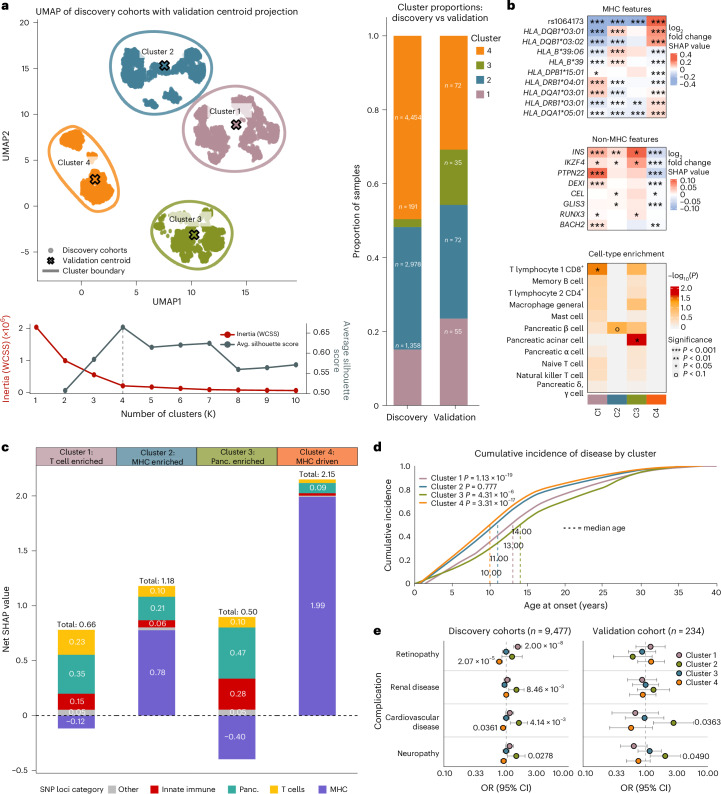
T1GRS reveals clinically relevant genetic subtypes of T1D. **a**, ScanPy-derived UMAP of SHAP values from the discovery cohort. Unsupervised clustering resulted in four distinct genetic clusters, determined based on optimal silhouette and inertia scores (see the plot below; dashed line indicates optimal cluster number). T1D individuals from the validation cohort in AoU were projected onto these existing clusters, with the centroid of individuals in each cluster shown. The membership proportion of T1D patients for each cluster is shown on the right. **b**, Per-cluster log_2_ fold-change of mean SHAP values relative to other clusters for candidate MHC (top) and non-MHC (middle) loci. *P* values were calculated by a two-sided *t* test. Cluster IDs are shown in the bottom annotation. Enrichment of cell-type regulatory elements for high-feature-importance loci in each cluster. *P* values were calculated by a permutation test. **c**, Total SHAP values for T1D individuals in discovery cohorts, broken down by features assigned to the MHC locus or specific cell types, split by cluster. **d**, Cumulative incidence of T1D for each cluster by age. *P* values calculated by log-rank test: cluster 1 *P* = 1.13 × 10^−19^, cluster 2 *P* = 0.777, cluster 3 = 4.31 × 10^−6^ and cluster 4 *P* = 3.31 × 10^−17^. Dashed lines indicate the median age of onset for each cluster. **e**, OR of clinical complications for the discovery dataset and AoU validation cohort split by cluster. *P* values were calculated by a two-sided Cox proportional hazards test. Error bars represent the 95% confidence intervals. Avg., average; CI, confidence interval; panc., pancreas.

Finally, we examined heterogeneity in the genetic factors driving T1D across individuals. We performed principal components analysis (PCA) of SHAP values from the T1GRS-vars model for all 29,746 individuals used to develop T1GRS, created a k-nearest neighbor graph from PCs and performed Leiden clustering. We then visualized the resulting clusters using dimension reduction of the PCs with Uniform Manifold Approximation and Projection (UMAP; Fig. 6a). We identified four subclusters of individuals based on T1GRS feature importance, where the optimal cluster number was defined based on inertia and silhouette score (Fig. 6a). Clusters were not dependent on specific cohorts or population structure (Supplementary Fig. 7a–d and Supplementary Table 15). We mapped an independent set of T1D individuals from AoU to these clusters, which had low centroid distances and similar proportions across clusters (Fig. 6a and Supplementary Table 16).

We next characterized the properties of each subcluster. We identified variants with significantly higher feature importance in each cluster (Fig. 6b and Supplementary Table 17). For example, HLA class II alleles were most important to cluster 4 and HLA class I alleles were most important to cluster 2, while the *PTPN22* locus was most important to cluster 1 and the *INS* locus to cluster 3. Second, we tested variants most important to each cluster for enrichment in *cis*-regulatory elements within T1D-relevant cell types, which revealed enrichment of cluster 1 for T cells and clusters 2 and 3 for pancreatic cells (Fig. 6b and Supplementary Table 18). Finally, we categorized variants mapping to the MHC locus or to loci linked to relevant cell types and calculated the cumulative SHAP contribution of categories to each cluster (Fig. 6c and Supplementary Table 19). Cluster 4 and to a lesser extent cluster 2 had the strongest contributions from MHC alleles, while cluster 1 had the strongest enrichment for T cell loci and cluster 3 for pancreatic loci. Based on these collective results, we renamed clusters ‘MHC-driven’ (cluster 4), ‘MHC-enriched’ (cluster 2), ‘T cell-enriched’ (cluster 1) and ‘pancreas-enriched’ (cluster 3). Variants with higher feature importance in the ‘pancreas-enriched’ cluster were also enriched for T2D loci (OR = 2.2), although this estimate was not significant (*P* > 0.05), with less enrichment in other clusters (Supplementary Table 19; Methods).

We next examined the risk of developing T1D for individuals within each genetic subcluster. Differences were observed in the average T1GRS scores across clusters—the two MHC clusters had the highest T1GRS scores and the pancreas-enriched cluster had the lowest scores. The segregation of T1D from nondiabetic individuals, however, was consistent and significantly different across all four clusters (Supplementary Fig. 7e). We next investigated T1GRS scores within each cluster separated by MHC and non-MHC variants. The pancreas-enriched cluster had lower MHC scores but comparable non-MHC scores relative to other clusters. Notably, all clusters were significantly separated between T1D and nondiabetes in both the MHC and non-MHC variant components (Supplementary Fig. 7e).

Finally, we determined whether genetic subclusters had differences in clinical characteristics relevant to T1D. There was a significant difference in T1D age of onset across clusters, with the MHC-related clusters showing an average onset several years earlier than the other clusters (Fig. 6d). We also observed significant differences in the rate of diabetes-related complications across clusters. Despite having the latest age of onset, individuals in the pancreas-enriched cluster had significantly higher rates of complications, including nephropathy (1.29-fold), neuropathy (1.35-fold) and cardiovascular disease (CVD; 1.46-fold; Fig. 6e) with no corresponding difference in HbA1c level (Supplementary Fig. 7f and Supplementary Table 20). The increased prevalence of diabetes-related complications in the pancreatic cluster was replicated in AoU for nephropathy (1.23-fold), neuropathy (1.44-fold) and CVD (2.34-fold; Fig. 6e).

Overall, these results reveal genetic subclusters that are significantly associated with the timing and likelihood of developing T1D and its complications.

## Discussion

We developed a model named T1GRS that can predict T1D using genetic data, which shows improved accuracy in European ancestry individuals compared to previous T1D risk scores^16,17^. T1GRS particularly improved predictions in individuals with fewer large-effect HLA haplotypes and with more complex contributions to genetic risk. While T1D risk scores are often benchmarked using highly curated T1D cohorts with enriched family history and early age of onset^16,17^, these individuals represent only a subset of T1D. When applied to population-based cohorts, which likely reflect a broader spectrum of T1D cases, the improvement of T1GRS over previous scores was more pronounced. Although trained on European ancestry individuals, T1GRS also had the same predictive ability for T1D in African Americans as a GRS specifically designed for this population^28^. Overall, these results demonstrate that this risk score can accurately predict T1D across diverse individuals and ancestries, supporting its wider applicability in both research and clinical settings. Future studies that train similar machine learning models using fine-mapping of shared and ancestry-specific variants through trans-ethnic studies^28^ will likely further improve the prediction of T1D across diverse ancestries.

The results of our study also provide an extensive collection of T1D risk signals and putative causal variants, which together will inform mechanistic studies to understand the drivers of T1D. Multiple new loci were localized to immune- and/or β-cell-related genes such as *ZMIZ1*, *CD6* and *CLNK*. New risk signals at the MHC locus revealed noncoding risk at this locus, which may help reveal specific cell type(s) and contexts through which class I and class II HLA genes influence disease. In addition, our model revealed extensive epistasis between T1D risk variants at the MHC locus and genome-wide, which provides potential insight into the mechanistic basis underlying T1D association. For example, the significant interaction between the *INS* locus and *HLA-DQB1* suggests that variation in the insulin gene may modulate risk of T1D through its relationship with class II MHC complexes. Genome-wide, the strongest interaction was observed between the *TOX* and *DEXI* loci, both of which have been implicated in β-cell function and may interact to affect β-cell survival^29,30^. Although the strongest interactions appeared to involve multiplicative effects, it is possible that interactions between T1D variants also involve dominant or recessive effects.

Grouping individuals based on features from T1GRS revealed genetic subclusters with significant heterogeneity in clinical features, such as age of onset and risk of diabetes-related complications. Recent studies have argued for the existence of endotypes of T1D characterized by age of onset, degree of insulitis and β-cell destruction and proinsulin processing^31^. These endotypes were defined using tissue from a few donors and relatively few islets per donor and the extent to which these patterns generalize is unclear. Furthermore, many of these measurements are not feasible to collect from living donors, limiting clinical value. Other recent evidence has supported the existence of heterogeneity in genetic risk of T1D, for example, dependent on age of onset or HLA background^32,33^. Our study reveals more extensive genetic heterogeneity in T1D, with direct relevance to its clinical management. Given the ability to obtain genetic profiles from all individuals, these subclusters could help guide clinical practice for T1D.

A key goal of this study was to provide a predictive model of T1D that was highly accessible to the clinical and research community. Our model uses only variants present on TOPMed r3 and the Michigan HLA reference panel, which can be accessed through public web tools. By comparison, previous approaches used proxy variants to capture HLA alleles that may not be present in reference panels^16,17^. Furthermore, using proxy SNPs to phase HLA alleles leads to ambiguity in HLA typing for some individuals, including those with DR3/DR4 haplotypes who are at greatest risk for T1D. By comparison, the T1GRS model uses variants directly from imputation panels for risk prediction, therefore avoiding issues with ambiguous phasing. We facilitated the prediction of T1D using T1GRS by providing containerized scripts that can be easily run in any computing environment.

Our study has several limitations for future studies to address. First, while population-based biobanks provide access to a broader spectrum of T1D individuals, an accurate definition of T1D from electronic health records (EHRs) and laboratory measurements remains a challenge^34^. Second, as T1D is a complex disease with both genetic and environmental components, there are inherent limitations to the predictive ability of genetic data. Composite models that combine genetic data with molecular features reflecting environmental exposures will help further improve T1D prediction^3,19^. In addition, as the specific environmental triggers of T1D remain unknown^35^, their identification would represent a major advance in the prediction of T1D. Third, our association study doesn’t extensively consider rare risk variants, which may further improve prediction^36^.

In total, T1GRS should facilitate and improve the use of GRS for T1D in both basic research and clinical applications. More broadly, our results highlight the value of combining the results of genetic association studies with machine learning methods to improve the prediction of complex diseases.

## Methods

### Ethics statement

The use of human genetic data in this study was approved by the University of California, San Diego, Institutional Review Board.

### Genome-wide association and genotype imputation

For the MHC analysis, we compiled genotype data from 10,107 T1D and 19,639 nondiabetic individuals of European ancestry across five cohorts (Supplementary Table 1). Cohorts were selected based on the availability of genome-wide genotyping array data for imputation into the TOPMed r3 panel^24,37,38^ and the MHC locus using the Michigan HLA reference panel^39^ and several cohorts (GENIE ROI, CSGNM) were further excluded due to lower quality imputation at this locus. T1D cases were matched to nondiabetic individuals by ancestry and, where possible, genotype array^9^. We performed quality control on variants using the HRC imputation preparation program (v4.2.9, https://www.well.ox.ac.uk/~wrayner/tools/) and PLINK (v1.9)to remove variants with MAF < 1%, missing genotypes >5%, violating Hardy-Weinberg equilibrium (HWE, *P* < 1 × 10^−5^ in unaffected cohorts; HWE, *P* < 1 × 10^−10^ in case cohorts), allele ambiguity and difference in allele frequency >0.2 compared to HRC r1.1 reference panel^38,40^. We imputed 55,615 variants from the four-digit multi-ethnic HLA reference panel (v1). We retained variants with imputation accuracy (*r*^2^) > 0.5 and a standard deviation in nondiabetic allele frequency < 0.055 for association testing^41^.

To examine genetic risk outside the MHC, we compiled association data from 20,355 T1D cases and 797,363 nondiabetic European ancestry individuals, matched by ancestry and genotype array where possible (Supplementary Table 11). For the FinnGen cohort, we downloaded the summary statistics from the r10 version of ‘T1D_Early’, which includes 2,832 individuals diagnosed with T1D under the age of 20 years and excludes individuals with T2D.

We used genotyping data for 263 individuals from nPOD, including 115 T1D cases and 148 individuals without T1D^42^. Additionally, we used 1,999 T2D individuals from the WTCCC1 cohort^43^. To examine the predictive ability of T1GRS in individuals of African ancestry, we used 284 T1D individuals from SEARCH and 404 nondisease individuals from CLEAR^44,45^. For all cohorts, we performed variant quality control as described above before imputation using the Michigan HLA and TOPMed r3 panels.

We considered participants with whole-genome sequencing and EHR data from the AoURP Controlled Tier Dataset v7 (ref. ^46^). T1D and nondiabetic individuals were identified using a combination of diagnosis codes (ICD-9/ICD-10) and drug exposures extracted from the EHR data. Briefly, a participant was classified as having T1D if they met all of the following criteria: (1) an ICD-9/ICD-10 diagnosis code for T1D on at least three visits or self-reported T1D on enrollment survey; (2) three or more instances of a recorded insulin prescription; (3) did not have a diagnosis of cystic fibrosis, secondary diabetes mellitus and drug- or chemical-induced diabetes mellitus; and (4) were not prescribed any other oral or injectable hypoglycemic agents. A participant was identified as nondiabetic if they did not have any of the following: (1) an ICD-9/ICD-10 code corresponding to T1D/T2D or self-reported T1D, or (2) an insulin prescription. Diagnosis codes used for classification were as follows: T1D—ICD-9-CM 250.x1, 250.x3; ICD-10-CM E10.x; T2D—ICD-9-CM 250.x0, 250.x2; ICD-10-CM E11.x; cystic fibrosis—ICD-9-CM 277.00–277.09; ICD-10-CM E84.x; secondary diabetes mellitus—ICD-9-CM 249.x and ICD-10-CM E08.x, E13.x; and drug-induced or chemical-induced diabetes mellitus—ICD-10-CM E09.x.

For insulin drug exposures, we included all medications classified under the ATC code A10A, including fast, intermediate, long, combination and inhaled insulins. Noninsulin hypoglycemic agents were defined by ATC code A10B and include biguanides, sulfonylureas, α-glucosidase inhibitors, thiazolidinediones, dipeptidyl-4 inhibitors, glucagon-like peptide 1 analogs, sodium-glucose cotransporter 2 inhibitors, meglitinides and their combinations.

Complications were defined by the presence of an ICD-9/ICD-10 code related to the complication. Cardiovascular complications were defined as myocardial infarction, cerebral infarction, heart failure, major vessel atherosclerosis, renal disease identified as a diagnostic code of diabetes and any severity of renal disease or separate ICD-9/ICD-10 code of proteinuria. Neuropathy was defined as any diagnostic code listing diabetes and any severity of neurologic complication. Retinopathy was defined using the following codes: cardiovascular—ICD-9-CM 410.7, 410.1x, 410.4x, 410.6x, 410.9x, 410.9, 428.xx, 434.x1, 437.0, 440.0, 440.1, 440.8; ICD-10-CM 121.x, 150.x, 163.x, 167.2, 170.0, 170.1, 170.8; nephropathy—ICD-9-CM 249.4x, 250.4x, 791.0; ICD-10-CM E08.2x, E09.2x, E10.2x, E11.2x, E13.2x, R80.x, N06.x; neuropathy—ICD-9-CM 250.6x, 249.6x; ICD-10-CM E08.4x, E09.4x, E10.4x, E11.4x, E13.4x; and retinopathy—ICD-9-CM 362.0x and 250.5x; ICD-10-CM E08.3x, E09.3x, E10.3x, E11.3x, E13.3x.

Details of the generation and quality control of the genomic data are provided in the AoURP Genomic Quality Report, release C2022Q4R9. Briefly, we used computed genetic ancestries provided by AoU to identify participants of European Ancestry. Genomic quality control and imputation were performed as described above for research participants.

### Association testing and meta-analysis

We used the first bias-corrected LogReg in EPACTS (v3.3.0, https://genome.sph.umich.edu/wiki/EPACTS) and variants for association with MAF > 1% at the MHC locus and MAF > 0.1% genome-wide were tested, including the first four genotype PCs and sex as covariates. In the MHC locus, we tested variants for association across a 4 Mb locus on chromosome 6 spanning 30–34 Mb (hg19). For both MHC-specific and genome-wide association analyses, we combined summary statistics from all tested cohorts using a fixed-effects inverse variance-weighted meta-analysis. For new loci, we considered variants with *P* < 1 × 10^−8^, identified as a more appropriate significance threshold for MAF > 0.1% variants^47^. For replication analyses, we updated the meta-analysis by replacing the summary statistics for the ‘T1D_EARLY’ phenotype in Finngen with those from the more recent r12 version, which includes an additional 219 T1D cases and 409,349 nondiabetic individuals. From the meta-analysis, we estimated the degree of confounding using LD score regression^48^ by calculating the intercept from one (0.0844), which supported a minimal effect of residual population structure on the results.

### Conditional analysis of independent signals

To identify independent signals at the MHC locus, we performed stepwise conditional analyses by including the most significant variant from each meta-analysis as a covariate in the association tests for each cohort, followed by reperforming through meta-analysis. We repeated this process by iteratively adding each new variant to the model until no variants remained significant at *P* < 5 × 10^−8^, where this threshold was selected based on testing variants with MAF > 1%. We also performed a ‘preconditional’ analysis to examine the effect of signals outside of 70 established class I and II HLA risk alleles and 20 DR3/DR4 pairwise HLA haplotypic and allelic interactions^24,49–51^. We added 90 covariates into the model to capture the additive effect of each alternate allele across 70 HLA risk alleles, and a binary column for each of the 20 interactions, before performing association testing and meta-analysis. We then performed stepwise conditional analyses by iteratively adding the most significant variant as an additional covariate in the model and reperforming the meta-analysis until no variants remained significant at *P* < 5 × 10^−8^.

### Credible set generation for signals in the MHC

For all independent signals identified at the MHC locus through stepwise conditional analysis, we generated 95% credible sets. We first identified variants in linkage with the lead variant for each signal (*r*^2^ > 0.1) and we calculated the Bayes Factor for each variant based on the effect and standard error as described by Wakefield^52^. We generated PIP scores by dividing the Bayes Factor by the total sum of the Bayes Factors for all variants in the set. We included variants up to the 95% threshold in each credible set.

### SuSiE fine-mapping of the non-MHC loci

We used SuSiE (v0.11.42) to fine-map loci identified through two rounds of variant clumping using PLINK (v1.9; ‘--clump-p1 5e-8 --clump-p2 0.05 --clump-r2 0.1 --clump-kb 10000’; ‘--clump-p1 5e-8 --clump-r2 0 --clump-kb 500’). We generated loci 500 kb around the variants in each clumped region, considering all variants regardless of MAF. We identified 87 significant loci and included 10 additional known loci not reaching genome-wide significance (*CDKN1C*, *CYP27B1*, *LMO7*, *CCR7*, 17q24, *ACOXL*, *CCR5*, *IRF2*, *TAGAP*, 6q27) for a total of 97 loci. We created 95% credible sets for each locus in SuSiE using genotypes from six dbGAP cohorts, including 32,518 individuals (DCCT, GENIE ROI, GENIE UK, GoKIND, T1DGC and WTCCC1) to define the LD matrix and set parameters to ‘*L* = 10, coverage = 0.95, min_abs_corr = 0.01, max_iter = 50,000’. For complex loci with multiple signals identified in a previous study (*DLK1*, *IFIH1*, *TYK2*, *IL10*, *PTPN2*, *AIRE*, *UBASH3A*, *CTLA4*, *IL2RA*), we recomputed the meta-analysis using only the six dbGAP cohorts with genotype data included in the LD matrix above^9^. Lead variants were defined as the variant with the largest PIP for the signal. We defined new loci as variants that reached genome-wide significance and mapped > 500 kb from other known loci.

### Annotations of credible sets

We leveraged genomic datasets to examine preferential TF binding to annotate new credible sets. We overlapped all credible set variants with accessible chromatin peaks in 46 immune and 12 pancreatic cell types^26,53^. We tested each variant for preferential allelic binding to TF motifs using FIMO (v4.12.0)^54^. We also leveraged databases such as GTEx and JASPAR to annotate credible set variants^55,56^.

### Constructing a nonlinear machine learning polygenic risk score

We leveraged 199 variants, including the lead variants from 27 MHC signals, 70 established HLA-associated alleles and 102 non-MHC lead variants (including five putative loci). We developed two models based on the CatBoost classifier framework (v1.0.6), one with the 199 variants alone (‘T1GRS-var’ model) and another with additional covariates of sex, PC1-4 and binary covariates for each cohort (‘T1GRS-cov’ model). This approach generates a probability ranging from 0 to 1 that represents the model’s confidence that an individual has T1D, which can be treated as a GRS. We refer to this probability as a ‘score’ to avoid confusion that it represents the actual probability of T1D. The discovery dataset, comprising five cohorts with 10,107 T1D and 19,639 unaffected individuals, was combined into a single genotype matrix, which was randomly split into ten subsections for cross-fold validation. Across ten iterations, a model was trained on 90% of the data and evaluated on the remaining 10% (as outlined previously^57^). Hyperparameters were determined by exhaustive grid search on the first cross-validation fold of the discovery dataset. Briefly, we used a binary CatBoostClassifier with 254 estimators, a depth of 5, a learning rate of 0.12 and a gradient boosting method. Specific hyperparameter settings are available at https://github.com/Gaulton-Lab/t1d-grs-analysis-catboost.

The probability scores for individuals in each testing fold were recorded and used to calculate the overall AUC of the model. This process was identical in the T1GRS-cov and T1GRS-var models, including all variant, MHC-only and non-MHC submodels. A representative model for each evaluation was trained on all individuals for validation purposes. Independent validation was performed on the NIH AoU Research Cohort containing 234 T1D and 78,658 nondiabetic individuals and the nPOD cohort, comprising 115 T1D and 148 nondiabetic individuals. A standard random seed was set to ensure reproducibility and a frozen model with identical hyperparameters was used for every validation.

### Generation of a LogReg comparison model

As a comparison to our T1GRS model, we also built a LogReg classifier. This model learns a single weight for each feature and performs a linear combination to predict the probability of T1D. The model outputs a probability score between 0 and 1. To prevent overfitting and allow the model to learn weaker non-MHC-based predictors of T1D, we applied strong L2 regularization (*C* = 0.001). The LogReg model was trained and evaluated using the same tenfold cross-validation format as all T1GRS models, using the same seed and training data.

### Feature importance and interaction analysis

Feature importance and interaction within nonlinear models were calculated using the SHAP machine learning interpretability suite (v0.41.0)^58^. SHAP is an approach to explain the output of any machine learning model based on cooperative game theory and the concept of Shapley values. SHAP values assign each feature an importance value for a particular prediction in the context of a specific model. The magnitude of feature importance is determined by the mean absolute value of all SHAP values for a given feature. SHAP values also capture nonlinear interactions between features on a per-individual basis and enable ranking pairwise feature interactions by magnitude. Each model was run through the standard SHAP pipeline and feature importance was recorded. Feature interaction analysis was performed using the shap_interaction_values function. To identify significant feature interactions, we converted all pairwise interaction values to *z* scores and calculated *P* values for each interaction using a *z* test. We then applied FDR correction to *P* values.

### Complexity scores

To understand how complex a model’s decision is for an individual’s disease classification, we developed a ‘complexity’ score, which is the total displacement of SHAP values for an individual, calculated by summing the absolute values of each feature, resulting in a single score per individual. A lower complexity score suggests a more straightforward classification, for example, driven by one or a few highly influential factors. For example, an individual with a strong MHC signal as the primary driver for a positive classification and minimal influence from other non-MHC features would likely exhibit a low score. Conversely, a higher complexity score indicates that many features, each contributing a smaller amount, collectively lead to the disease outcome. Individuals were assigned to deciles based on their complexity scores.

### Defining genetic subtypes and validation

Individual-specific SHAP feature contribution vectors from the T1GRS-var model formed the basis of this analysis for both discovery and validation cohorts. All analyses were made reproducible by using a fixed random seed. Starting with the discovery cohort, high-dimensional SHAP vectors were first reduced using PCA, retaining up to 175 principal components. We then employed high-dimensional clustering in ScanPy (v1.8.2)^59^ as follows: in PCA space, a *k*-nearest neighbor graph was constructed (using 120 neighbors). This graph was then used to generate a two-dimensional UMAP embedding, with a ‘min_dist’ parameter of 0.25. Subgroups of individuals within the discovery cohort were then identified by applying the Leiden community detection algorithm to the kNN graph, using a resolution of 0.05. Next, we used the ScanPy ingest workflow to project the validation cohort onto our existing clusters. To assign validation individuals to the clusters, PCA representations were projected into the discovery cohort’s UMAP space and then assigned to clusters using the same Leiden algorithm.

### Analysis of age of onset, clinical complications and T2D loci in T1D subtypes

To identify differences in age of onset between clusters, we performed a log-rank test and considered significant differences at *P* < 0.05. To identify differences in clinical complications between clusters, the OR was calculated in the discovery and AoU datasets and *P* values were calculated using a Cox proportional hazards test. To determine enrichment of T2D loci within clusters, we defined T1GRS variants in LD (*r*^2^ > 0.2) with reported T2D variants^60^. We performed LogReg for each cluster using mean-normalized SHAP values for each locus as the predictor and T2D association as the binary outcome.

### Analysis of T1D GRS

We calculated GRS2 using 60 exact TOPMed variants, 2 exact Michigan HLA for rs116522341, rs1281934, and the proxy variants DQB1*06:02, B*18:01, DPB1*03:01, rs1611547 and rs114170382 from Michigan HLA for rs17843689, rs371250843, rs559242105, rs144530872 and rs149663102, respectively. In GRS2, we excluded individuals with more than two HLA-DR/DQ proxy SNPs according to the published methods^16^. Within each GRS, we examined the total GRS and its components of MHC and non-MHC variants. We calculated the AUC for the receiver operating characteristic analysis to assess the differentiation power of each GRS for T1D. We then tested the differences between AUCs using the DeLong test. First, we compared T1GRS and GRS2 in individuals with T1D and those without using both the ‘T1GRS-cov’ and the ‘T1GRS-var’ models. Next, we validated T1GRS using individuals in the nPOD biorepository to differentiate between T1D and nondiabetes and using the T1D from nPOD and 1,999 T2D from WTCCC1 (ref. ^43^).

We calculated a published African ancestry risk score in 284 T1D and 404 nondiabetic individuals from SEARCH and CLEAR to compare with T1GRS^44,45^. We used TOPMed to impute rs34850435, rs9271594, rs9273363, rs2290400 and rs689, while Michigan HLA was used to impute rs2187668. The variant rs9268838 was used as a proxy for rs34303755 (*r*^2^ = 0.849, *D*′ = 1.0 in African ancestry).

Lastly, we generated a scale for T1GRS scores using the number of individuals with T1D at various percentiles and calculated a diagnostic for each GRS value using the Youden index (sensitivity + specificity − 1). We calculated sensitivity at each GRS score on the scale as TP/(TP + FN) and specificity as TN/(TN + FP)^61^. We defined DR3/DR4 individuals using four-digit HLA alleles imputed from the T1DGC reference panel using SNP2HLA^32^. DR3 status was classified as HLA-DRB1*03:01–DQB1*02:01, while DR4 as HLA-DRB1*04:01/*02*/*04*/*05*/08–DQB1*03:02/*04*/*02:02* (ref. ^33^).

### Variant category classification

To assign variants used in T1GRS to cell types, we first intersected credible sets with the cell-type atlas of *cis*-regulatory elements (CATlas)^53^ for pancreatic and immune cell types. For loci that did not intersect these *cis*-regulatory elements, we determined credible set intersection with gene bodies in GENCODE GRCh38.p14 (ref. ^62^) and annotated loci with cell types based on gene expression patterns. Lastly, for several loci previously linked to specific genes, we annotated loci to cell types based on the expression patterns of these genes. The full list of links between loci in T1GRS and cell types, as well as the references for the links, is provided in Supplementary Table 18.

### Intersection of cluster loci and cell-type regulatory elements

To identify the top loci for each cluster, SHAP values were averaged across all individuals within the cluster. For each T1GRS variant, SHAP values were normalized across clusters to sum to 1. We identified loci for each cluster with a normalized SHAP value greater than 0.75. Using the top loci for each cluster, credible sets for these loci were intersected with regulatory elements for 12 immune and pancreatic endocrine cell types derived from ENCODE. For each cluster, the posterior probability of association for all variants intersecting a regulatory element in a cell type was summed. Permutation testing was performed by shuffling the cumulative probability of associations across all cell types 10,000 times for each cluster and calculating a *P* value from this null distribution.

### Reporting summary

Further information on research design is available in the Nature Portfolio Reporting Summary linked to this article.

## Online content

Any methods, additional references, Nature Portfolio reporting summaries, source data, extended data, supplementary information, acknowledgements, peer review information; details of author contributions and competing interests; and statements of data and code availability are available at 10.1038/s41588-026-02578-y.

## Supplementary information

Supplementary Information Supplementary Note and Figs. 1–7.

Reporting Summary

Supplementary Tables Supplementary Tables 1–19.

## Data Availability

Summary statistics from the T1D GWAS are available in the GWAS catalog with accession GCST90824163 (https://www.ebi.ac.uk/gwas/studies/GCST90824163).

## Code availability

Custom code used to build the T1GRS model, predict risk scores and generate relevant plots can be found at Zenodo (10.5281/zenodo.18842258)^63^ and on GitHub (https://github.com/Gaulton-Lab/t1d-grs-analysis-catboost).
