# Supplementary material for: Genetic association and machine learning improve the prediction of type 1 diabetes risk
Source: Nat Genet. 2026 Apr 30;58(5):1062–72. doi: 10.1038/s41588-026-02578-y (PMC13175890; doi:10.1038/s41588-026-02578-y)
Supplement: Supplementary file 1 — Supplementary Note and Figs. 1–7. [file 41588_2026_2578_MOESM1_ESM.pdf]

nature genetics

Supplementary information https://doi.org/10.1038/s41588-026-02578-y

Genetic association and machine learning improve the prediction of type 1 diabetes risk

In the format provided by the authors and unedited

Supplementary Information

Table of contents:

Supplementary Text
Acknowledgements
Supplementary Figure 1
Supplementary Figure 2
Supplementary Figure 3
Supplementary Figure 4
Supplementary Figure 5
Supplementary Figure 6
Supplementary Figure 7

Supplementary text

Thirteen previously unreported loci reached a significance threshold of $P<5x10^{-8}$, where six reached a more stringent significance threshold of $P<1x10^{-8}$ and two more replicated at that same threshold after incorporating an additional 219 T1D cases and 87,948 non-T1D in Finngen (**Supplementary Table 2**). Therefore, we considered these eight unreported loci as novel T1D risk loci and the other five as putative loci that require further validation.

The predictive ability of T1GRS to discriminate T1D from T2D was largely driven by MHC variants (AUC=0.899) (**Supplementary Fig. 3F**) and to a lesser extent non-MHC variants (AUC=0.729) (**Supplementary Fig. 3G**). Compared to GRS2 applied to the same individuals, T1GRS-var had significantly improved prediction overall (GRS2 AUC=0.867, p=0.0457) and in non-MHC variants (GRS2 AUC=0.605, p<0.001), and marginal but not significant improvement in MHC-only variants (GRS2 AUC=0.867, p=0.185) (**Supplementary Fig. 3E-G**). As expected, the performance of T1GRS-var model was significantly reduced in African American relative to European ancestry individuals (EUR AUC=0.923, AA AUC=0.845, p<0.0001) (**Supplementary Fig. 3H**) with a significant reduction in both MHC (AA AUC=0.845, EUR AUC=0.894, p<0.0001) and non-MHC variants (AA AUC=0.584, EUR AUC=0.714, p<0.0001).

We investigated T1D predictions based on differences in the definition of MHC alleles in T1GRS and GRS2. In our discovery cohorts, a total of 978 individuals (630 T1D, 348 unaffected) had incompatible MHC class II allele status using the published GRS2 method leading to an inability to calculate a risk score for those individuals. In comparison, T1GRS uses variants imputed from the Michigan HLA reference panel and avoids these incompatibilities. Of the 630 T1D individuals with incompatible MHC class II alleles in GRS2, 64.1% (404) were classified above the 50th T1D percentile in T1GRS (**Supplementary Fig. 3M**). When examining 3,810 individuals from discovery cohorts with the HLA-DR3/DR4 haplotype (**see Methods**), which confers the highest genetic risk of T1D, 8.8% had HLA-DQ ambiguity in GRS2 resulting in no prediction for these individuals. In contrast, T1GRS scored 84% of these individuals above the 50th percentile, demonstrating an improved ability to predict T1D in a larger proportion of individuals with the highest genetic risk (**Supplementary Fig. 3M**). In the discovery cohort, 28% of individuals with a T1GRS score greater than the 50th percentile for T1D had a GRS2 score below the 50th percentile (GRS2=14.6) (**Supplementary Fig. 4B**). Among these individuals, 62% were above the 50th percentile in T1GRS using non-MHC variants.

We determined the potential value of T1GRS as a diagnostic. The Youden index can indicate diagnostic feasibility of a GRS when greater than 0.50. The maximum Youden index for T1GRS was 0.733 at a T1GRS score of 0.574, corresponding to 89% sensitivity and 84% specificity for T1D, which is an improvement over

the maximum Youden Index for GRS2 of 0.683 at the same sensitivity (89%) but lower specificity (79%). Previous T1D GRS have shown that at a 50% sensitivity for T1D the false positive rate is 3.3-6% and at this same sensitivity threshold T1GRS has a lower false positive rate (2.1%). Similarly, at a false positive rate of 5%, the sensitivity of T1GRS was 68% which represents a 30% increase in the number of accurately classified T1D individuals at this threshold compared to GRS2. At 25% sensitivity the false positive rate was also reduced compared to previous GRS, where only 0.5% (103/19,291) of non-T1D fell above this threshold. The lower end of prediction range was improved as well, where 95% sensitivity for T1D in T1GRS had a higher specificity of 73% compared to GRS2 (**Table 1, Supplemental Table 12**). The distribution of scores between T1D and non-T1D also had cleaner separation in T1GRS compared to GRS2 (**Supplementary Fig. 3D**).

Acknowledgements

DCCT/EDIC

The Diabetes Control and Complications Trial (DCCT) and its follow-up the Epidemiology of Diabetes Interventions and Complications (EDIC) study were conducted by the DCCT/EDIC Research Group and supported by National Institute of Health grants and contracts and by the General Clinical Research Center Program, NCRR. The data from the DCCT/EDIC study were supplied by the NIDDK Central Repositories.

GENIE

The Genetics of Nephropathy, an International Effort (GENIE) study was conducted by the GENIE Investigators and supported by the National Institute of Diabetes and Digestive and Kidney Diseases (NIDDK). The data from the GENIE study reported here were supplied by the GENIE investigators from the Broad Institute of MIT and Harvard, Queens University Belfast and the University of Dublin.

GoKinD

The Genetics of Kidneys in Diabetes (GoKinD) Study was conducted by the GoKinD Investigators and supported by the Juvenile Diabetes Research Foundation, the CDC, and the Special Statutory Funding Program for Type 1 Diabetes Research administered by the National Institute of Diabetes and Digestive and Kidney Diseases (NIDDK). The data from the GoKinD study were supplied by the NIDDK Central Repositories. This manuscript was not prepared in collaboration with Investigators of the GoKinD study and does not necessarily reflect the opinions or views of the GoKinD study, the NIDDK Central Repositories, or the NIDDK.

T1DGC

This research utilizes resources provided by the Type 1 Diabetes Genetics Consortium (T1DGC), a collaborative clinical study sponsored by the National Institute of Diabetes and Digestive and Kidney

Diseases (NIDDK), National Institute of Allergy and Infectious Diseases (NIAID), National Human Genome Research Institute (NHGRI), National Institute of Child Health and Human Development (NICHD), and the Juvenile Diabetes Research Foundation International (JDRF) and supported by U01 DK062418. The UK case series collection was additionally funded by the JDRF and Wellcome Trust and the National Institute for Health Research Cambridge Biomedical Centre, at the Cambridge Institute for Medical Research, UK (CIMR), which is in receipt of a Wellcome Trust Strategic Award (079895). The data from the T1DGC study were supplied by dbGAP. This manuscript was not prepared in collaboration with Investigators of the T1DGC study and does not necessarily reflect the opinions or views of the T1DGC study or the study sponsors.

T1DGC (ASP/UK GRID)

This research was performed under the auspices of the Type 1 Diabetes Genetics Consortium, a collaborative clinical study sponsored by the National Institute of Diabetes and Digestive and Kidney Diseases (NIDDK), National Institute of Allergy and Infectious Diseases (NIAID), National Human Genome Research Institute (NHGRI), National Institute of Child Health and Human Development (NICHD), and Juvenile Diabetes Research Foundation International (JDRF).

WTCCC

This study makes use of data generated by the Wellcome Trust Case Control Consortium. A full list of the investigators who contributed to the generation of the data is available from www.wtccc.org.uk. Funding for the project was provided by the Wellcome Trust under award 076113.

UK Biobank

Data from the UK Biobank was accessed under application 24058.

FinnGen

The FinnGen study is a large-scale genomics initiative that has analyzed over 500,000 Finnish biobank specimens and correlated genetic variation with health data to understand disease mechanisms and predispositions. The project is a collaboration between research organisations and biobanks within Finland and international industry partners. We want to acknowledge the participants and investigators of the FinnGen study.

CLEAR

The data used for the analyses described in this paper were obtained from the database of Genotypes and Phenotypes (dbGaP), at http://www.ncbi.nlm.nih.gov/gap. Genotype and phenotype data for the study “Genome-Wide Association Study in African-Americans with Rheumatoid Arthritis” were provided by Dr. S. Louis Bridges, Jr. University of Alabama at Birmingham. The GWAS study (R01AR057202) was supported

by awards from the National Institute of Arthritis and Musculoskeletal and Skin Diseases. The cohorts used in the GWAS study were derived from participants in the Consortium for the Longitudinal Evaluation of African-Americans with Early Rheumatoid Arthritis (CLEAR I and II) case-control observational studies, supported by a research contract awarded by the National Institute of Arthritis and Musculoskeletal and Skin Diseases. The consortium is a collaborative effort among five academic institutions: University of Alabama at Birmingham, Birmingham, AL (Coordinating Center); Grady Hospital/Emory University, Atlanta, GA; University of North Carolina, Chapel Hill, NC; Medical University of South Carolina, SC; and Washington University, St. Louis, MO. For specific publication describing the CLEAR studies, CLEAR collaborators, see https://www.uab.edu/medicine/rheumatology/research/70-clear and https://www.ncbi.nlm.nih.gov/pmc/articles/PMC3052790/.

SEARCH
The SEARCH for Diabetes in Youth study was conducted by the SEARCH Investigators and supported by the National Institute of Diabetes and Digestive and Kidney Diseases (NIDDK). The data from the SEARCH Study reported here were supplied by the institutions (funding) listed below:

Kaiser Permanente Southern California (U18DP006133, U48/CCU919219, U01 DP000246, and U18DP002714), University of Colorado Denver (U18DP006139, U48/CCU819241-3, U01 DP000247, and U18DP000247-06A1), Cincinnati's Children's Hospital Medical Center (U18DP006134, U48/CCU519239, U01 DP000248, and 1U18DP002709), University of North Carolina at Chapel Hill (U18DP006138, U48/CCU419249, U01 DP000254, and U18DP002708), Seattle Children's Hospital (U18DP006136, U58/CCU019235-4, U01 DP000244, and U18DP002710-01), Wake Forest School of Medicine (U18DP006131, U48/CCU919219, U01 DP000250, 200-2010-35171, DP15-0020301SUPP17, and 1UC4DK108173).

Network for Pancreatic Organ Donors with Diabetes (nPOD)
This research was performed with the support of the Network for Pancreatic Organ donors with Diabetes (nPOD; RRID:SCR_014641), a collaborative type 1 diabetes research project supported by JDRF (nPOD: 5-SRA-2018-557-Q-R) and The Leona M. & Harry B. Helmsley Charitable Trust (Grant#2018PG-T1D053). The content and views expressed are the responsibility of the authors and do not necessarily reflect the official view of nPOD. Organ Procurement Organizations (OPO) partnering with nPOD to provide research resources are listed at https://npod.org/for-partners/npod-partners/.

CSGNM
We thank the participants of the Trinity Student Study. This study was supported by the Intramural Research Programs of the National Institutes of Health, the National Human Genome Research Institute, and the Eunice Kennedy Shriver National Institute of Child Health and Development.

NIMH Schizophrenia Controls

Funding support for the Genome-Wide Association of Schizophrenia Study was provided by the National Institute of Mental Health (R01 MH67257, R01 MH59588, R01 MH59571, R01 MH59565, R01 MH59587, R01 MH60870, R01 MH59566, R01 MH59586, R01 MH61675, R01 MH60879, R01 MH81800, U01 MH46276, U01 MH46289 U01 MH46318, U01 MH79469, and U01 MH79470) and the genotyping of was provided through the Genetic Association Information Network (GAIN). The datasets used for the analyses described in this manuscript were obtained from the database of Genotypes and Phenotypes (dbGaP) found at http://www.ncbi.nlm.nih.gov/gap through dbGaP accession number phs000021.v3.p2. Genetic samples and associated phenotype data for the Genome-Wide Association of Schizophrenia Study were provided by the Molecular Genetics of Schizophrenia Collaboration (PI: Pablo V. Gejman, Evanston Northwestern Healthcare (ENH) and Northwestern University, Evanston, IL, USA).

Neurodevelopmental Genomics

Support for the collection of the data for Philadelphia Neurodevelopment Cohort (PNC) was provided by grant RC2MH089983 awarded to Raquel Gur and RC2MH089924 awarded to Hakon Hakonarson. Subjects were recruited and genotyped through the Center for Applied Genomics (CAG) at The Children's Hospital in Philadelphia (CHOP). Phenotypic data collection occurred at the CAG/CHOP and at the Brain Behavior Laboratory, University of Pennsylvania.

eMERGE Network

Group Health Cooperative/University of Washington – Funding support for Alzheimer's Disease Patient Registry (ADPR) and Adult Changes in Thought (ACT) study was provided by a U01 from the National Institute on Aging (Eric B. Larson, PI, U01AG006781). A gift from the 3M Corporation was used to expand the ACT cohort. DNA aliquots sufficient for GWAS from ADPR Probable AD cases, who had been enrolled in Genetic Differences in Alzheimer's Cases and Controls (Walter Kukull, PI, R01 AG007584) and obtained under that grant, were made available to eMERGE without charge. Funding support for genotyping, which was performed at Johns Hopkins University, was provided by the NIH (U01HG004438). Genome-wide association analyses were supported through a Cooperative Agreement from the National Human Genome Research Institute, U01HG004610 (Eric B. Larson, PI). Mayo Clinic – Genetic samples and associated genotype and phenotype data used in this study were provided by the Mayo Clinic. Funding support for the Mayo Clinic was provided through a cooperative agreement with the National Human Genome Research Institute (NHGRI), Grant #: UOIHG004599; and by grant HL75794 from the National Heart Lung and Blood Institute (NHLBI). Funding support for genotyping, which was performed at The Broad Institute, was provided by the NIH (U01HG004424). Marshfield Clinic Research Foundation – Funding support for the Personalized Medicine Research Project (PMRP) was provided through a cooperative agreement (U01HG004608) with the National Human Genome Research Institute (NHGRI), with additional funding

from the National Institute for General Medical Sciences (NIGMS) The genetic samples used for PMRP analyses were obtained with funding from Marshfield Clinic, Health Resources Service Administration Office of Rural Health Policy grant number D1A RH00025, and Wisconsin Department of Commerce Technology Development Fund contract number TDF FYO10718. Funding support for genotyping, which was performed at Johns Hopkins University, was provided by the NIH (U01HG004438). Northwestern University – Genetic samples and data used in this study were provided by the NUgene Project (www.nugene.org). Funding support for the NUgene Project was provided by the Northwestern University's Center for Genetic Medicine, Northwestern University, and Northwestern Memorial Hospital. Assistance with phenotype harmonization was provided by the eMERGE Coordinating Center (Grant number U01HG04603). This study was funded through the NIH, NHGRI eMERGE Network (U01HG004609). Funding support for genotyping, which was performed at The Broad Institute, was provided by the NIH (U01HG004424).

Vanderbilt University

Funding support for the Vanderbilt Genome-Electronic Records (VGER) project was provided through a cooperative agreement (U01HG004603) with the National Human Genome Research Institute (NHGRI) with additional funding from the National Institute of General Medical Sciences (NIGMS). The dataset and genetic samples used for the VGER analyses were obtained from Vanderbilt University Medical Center's BioVU, which is supported by institutional funding and by the Vanderbilt CTSA grant UL1RR024975 from NCRR/NIH. Funding support for genotyping, which was performed at The Broad Institute, was provided by the NIH (U01HG004424). Assistance with phenotype harmonization and genotype data cleaning was provided by the eMERGE Administrative Coordinating Center (U01HG004603) and the National Center for Biotechnology Information (NCBI). The datasets used for the analyses described in this manuscript were obtained from dbGaP at http://www.ncbi.nlm.nih.gov/gap through dbGaP accession number phs000360.v3.p1.

This manuscript was not prepared in collaboration with investigators of these studies and does not necessarily reflect the opinions or views of the DCCT/EDIC, GENIE, GoKinD, T1DGC, WTCCC, CLEAR, SEARCH studies or study groups, the NIDDK Central Repositories, the NIH, or the study sponsors.

All of Us

This research was conducted using data from the All of Us Research Program, a program supported by the National Institutes of Health. The All of Us Research Program is supported by the National Institutes of Health, Office of the Director, and through the NIH Common Fund.

Supplementary Figures

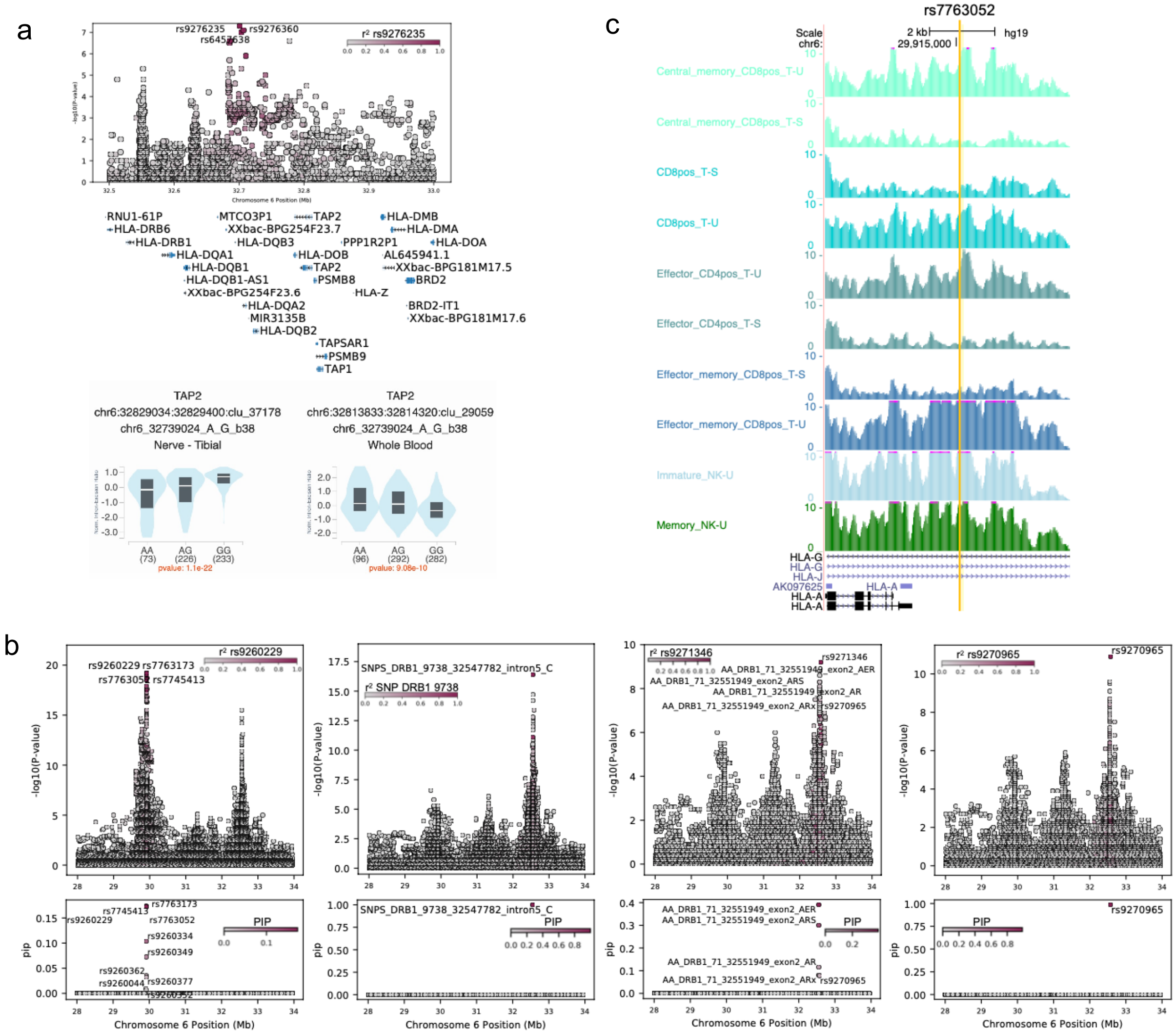

Supplementary Figure 1. Annotation of novel T1D signals at the MHC locus. (a) Locus plot showing T1D association p-values at a novel non-coding signal at the MHC locus with index variant rs9276235. P-values from meta-analysis of T1D association statistics from two-sided firth bias-corrected logistic regression of *n*=29,746 (10,107 T1D, 19,639 non-diabetic) individuals. Locus plots are colored by linkage (r^2) to the lead variant for each signal. The bottom of the plot shows expression QTLs for TAP2 at this variant from the GTEx database for multiple tissues. (b) Locus plots of p-values and credible sets for novel signals identified in pre-conditioned HLA T1D association analysis. P-values from conditional analyses using same methods and sample size as panel a. Colors represent linkage (r^2) with lead variant for each signal. (c) Annotation of a novel T1D signal upstream of HLA-A where variant rs7763052 maps (orange line) in accessible chromatin sites for stimulated (S) and unstimulated (U) conditions of different immune cell types.

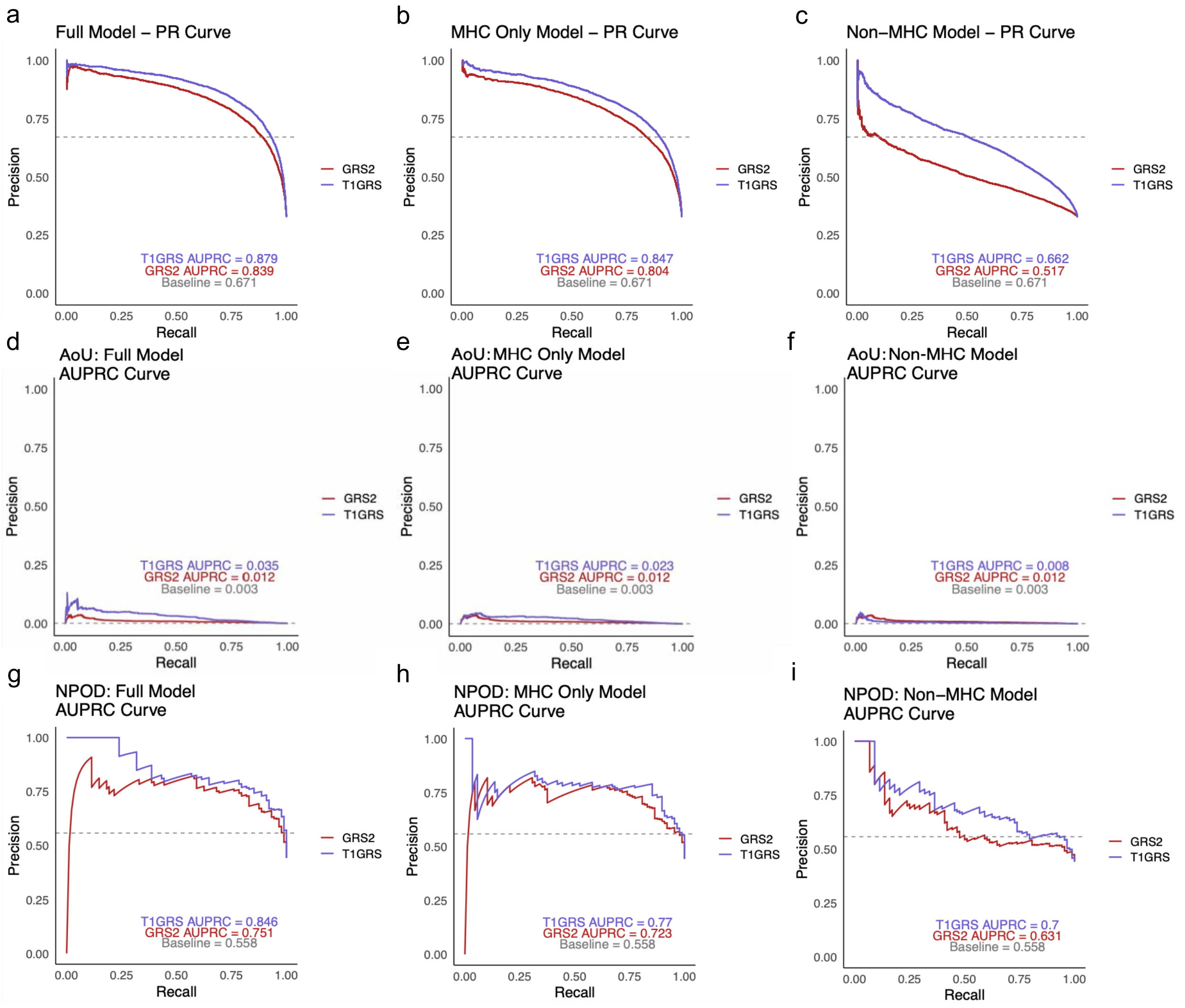

Supplementary Figure 2: Precision-Recall Curves in T1GRS and GRS2. Precision-Recall curves highlighting the Average Precision (AP) in T1GRS and GRS2, baselines indicated by dashed line. AP curves using discovery cohorts for (a) T1GRS-cov vs GRS2, (b) MHC Sub-model of T1GRS-cov vs MHC Variants in GRS2, and (c) Non-MHC Sub-model of T1GRS-cov vs non-MHC variants in GRS2. AP Curves using NIH All of Us Cohort for (d) T1GRS-var vs GRS2, (e) MHC Sub-model of T1GRS-var vs MHC variants in GRS2, and (f) Non-MHC Sub-model of T1GRS-var vs non-MHC variants in GRS2. AP Curves using NIH NPOD Cohort for (g) T1GRS-var vs GRS2, (h) MHC Sub-model of T1GRS-var vs MHC variants in GRS2, and (i) Non-MHC Sub-model of T1GRS-var vs non-MHC variants in GRS2.

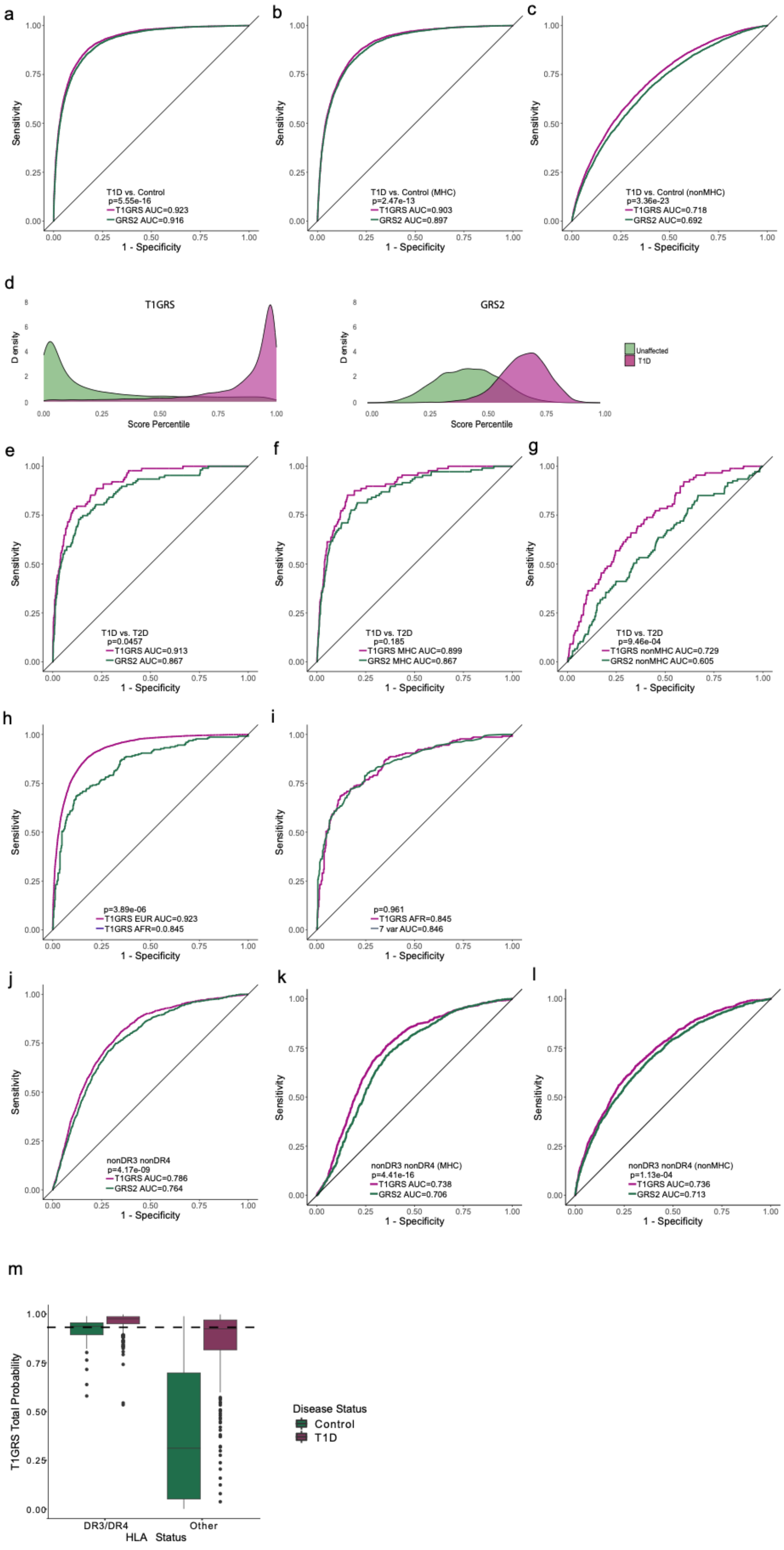

a
T1D vs. Control
p=5.55e-16
T1GRS AUC=0.923
GRS2 AUC=0.916
b
T1D vs. Control (MHC)
p=2.47e-13
T1GRS AUC=0.903
GRS2 AUC=0.897
c
T1D vs. Control (nonMHC)
p=3.36e-23
T1GRS AUC=0.718
GRS2 AUC=0.692
d
T1GRS
GRS2
Density
Score Percentile
Unaffected
T1D
e
T1D vs. T2D
p=0.0457
T1GRS AUC=0.913
GRS2 AUC=0.867
f
T1D vs. T2D
p=0.185
T1GRS MHC AUC=0.899
GRS2 MHC AUC=0.867
g
T1D vs. T2D
p=9.46e-04
T1GRS nonMHC AUC=0.729
GRS2 nonMHC AUC=0.605
h
p=3.89e-06
T1GRS EUR AUC=0.923
T1GRS AFR=0.0.845
i
p=0.961
T1GRS AFR=0.845
7 var AUC=0.846
j
nonDR3 nonDR4
p=4.17e-09
T1GRS AUC=0.786
GRS2 AUC=0.764
k
nonDR3 nonDR4 (MHC)
p=4.41e-16
T1GRS AUC=0.738
GRS2 AUC=0.706
l
nonDR3 nonDR4 (nonMHC)
p=1.13e-04
T1GRS AUC=0.736
GRS2 AUC=0.713
Sensitivity
1 - Specificity
m
T1GRS Total Probability
HLA Status
DR3/DR4
Other
Disease Status
Control
T1D

Supplementary Figure 3: T1GRS and GRS2 Comparative Model Performance. Receiver Operating Characteristics (ROC) comparing T1GRS vs GRS2 in discovery cohorts for (a) T1GRS-var vs GRS2, (b) MHC sub-model of T1GRS-var vs MHC variants and (c) non-MHC sub-model of T1GRS-var vs non-MHC variants in GRS2. P-values for panels a-c calculated using two-sided DeLong test. (d) Density distributions of scores output from T1GRS-cov and GRS2. ROC comparing T1GRS to GRS2 in T1D vs T2D individuals for (e) T1GRS-var vs GRS2, (f) MHC sub-model of T1GRS-var vs MHC variants GRS2, (g) non-MHC sub-model of T1GRS-var vs non-MHC variants in GRS2, (h) ROC comparing T1GRS-var predictive performance in T1D cases of European vs African ancestry, and (i) ROC comparing T1GRS-var vs previously published 7-variant African ancestry T1D GRS. ROC curve comparing T1GRS to GRS2 in individuals without HLA-DR3 or HLA-DR4 haplotypes for (j) T1GRS-var vs GRS2, (k) MHC sub-model of T1GRS-var vs MHC variants GRS2, and (i) non-MHC sub-model of T1GRS-var vs non-MHC variants in GRS2. P-values for panels e-l calculated using two-sided DeLong test. (m) T1GRS-cov score in individuals with HLA-DQ proxy SNP ambiguity in T1D GRS2 separated by high-risk HLA-DR3/DR4 (left) and all other HLA alleles (right). Box-and-whisker plots show median percentile and interquartile range.

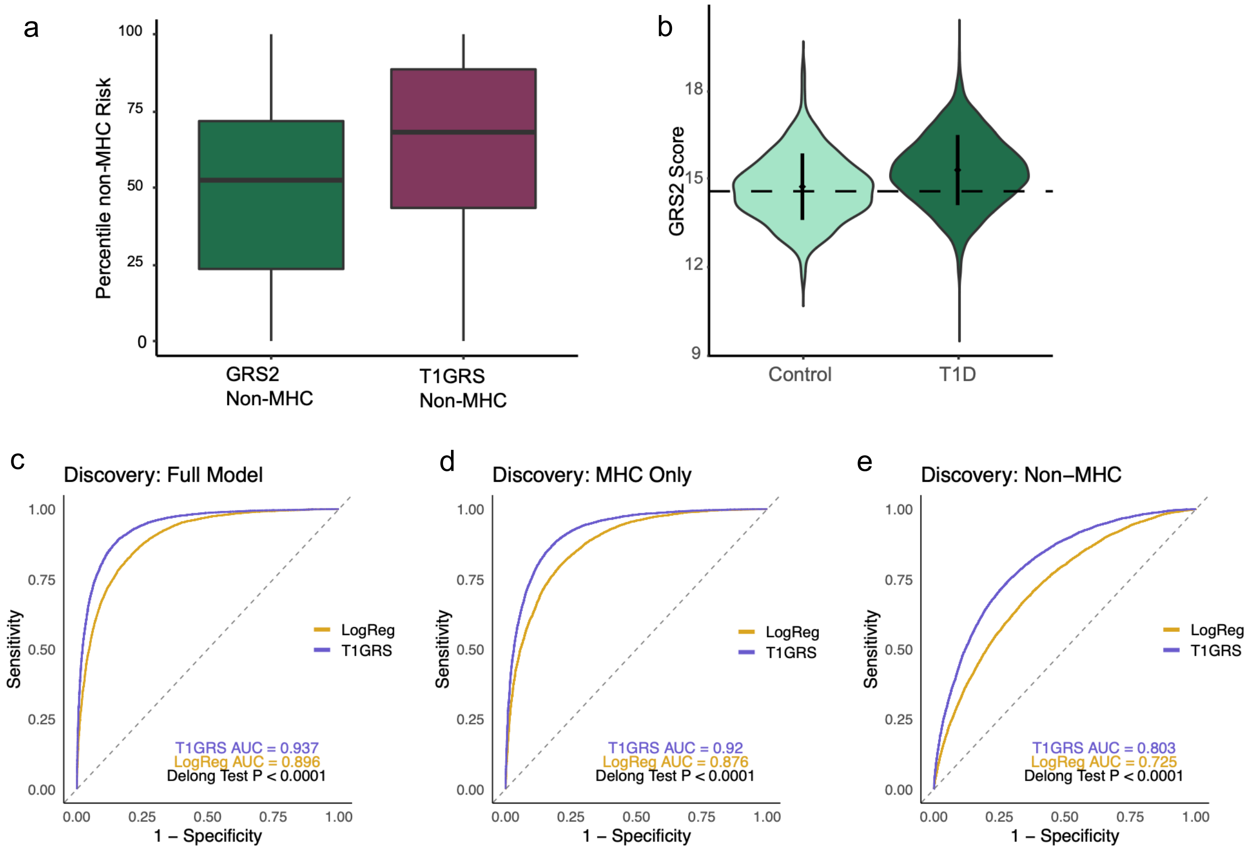

Supplementary Figure 4. T1GRS Improves Performance over GRS2 Non-MHC Variants and Interactions. (a) GRS2 and T1GRS-cov non-MHC percentiles for 157 T1D individuals with a large effect CEL risk variant. Box-and-whisker plots show median percentile and interquartile range. (b) Distribution of GRS2 risk scores for individuals above the T1D 50th percentile for T1GRS. Bars on plots show interquartile range. (c-e) ROC curves comparing classification of T1D using (c) T1GRS-cov and a logistic regression model of the same variants, (d) MHC-only sub-model of T1GRS-cov and a logistic regression model of the same variants, and (e) non-MHC sub-model of T1GRS-cov and a logistic regression model of the same variants. P-values calculated using two-sided DeLong test.

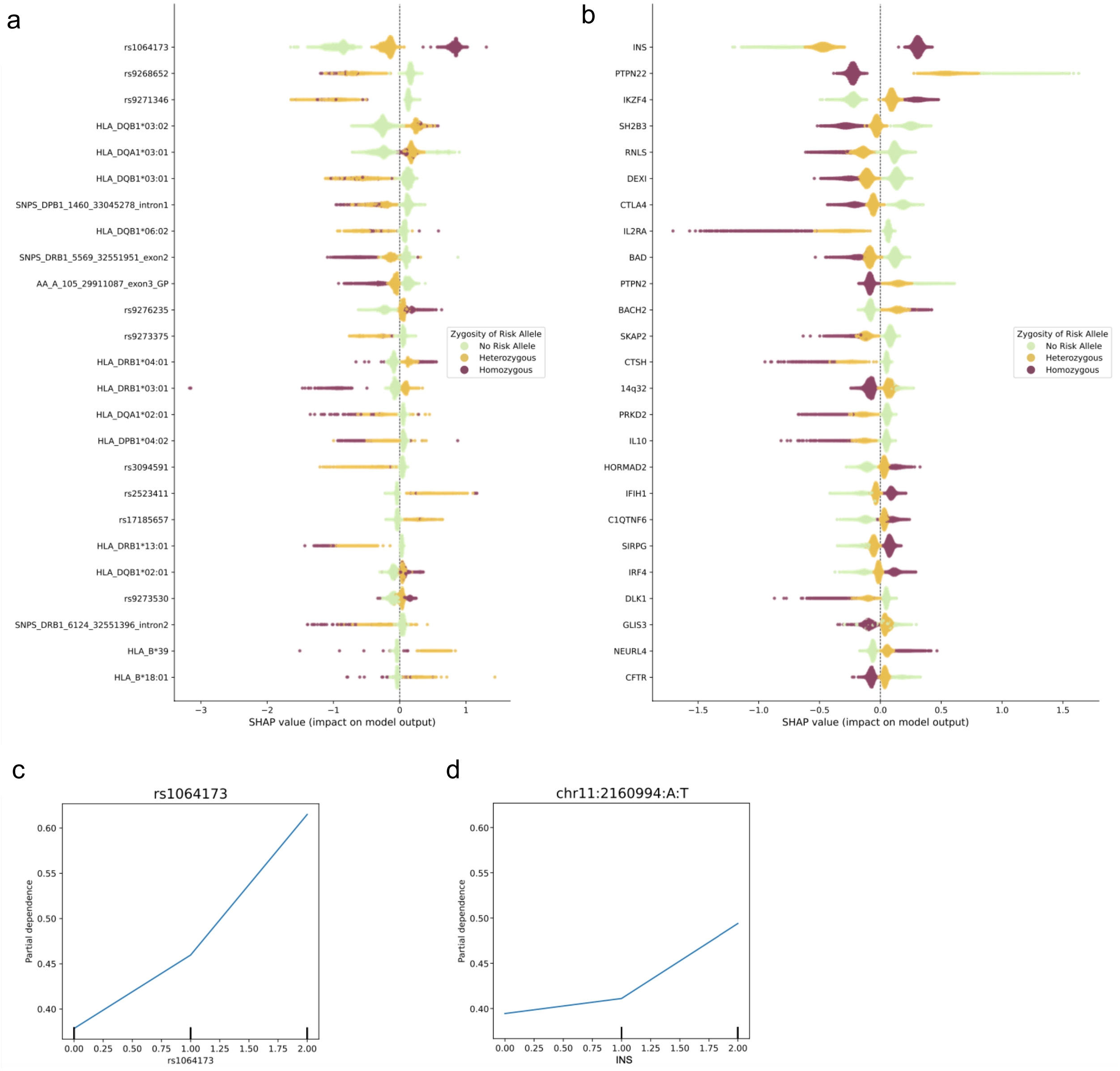

Supplementary Figure 5. Characterization of Interactions in T1GRS. (a) SHAP analysis results of feature importance in the top 25 MHC features for T1GRS-cov and (b) SHAP analyses in top 25 non-MHC features for T1GRS-cov. Colors in A, B indicate the contribution of 2,1, or 0 copies of the risk allele. Larger values on the x-axis indicates greater impact on model classification. Partial dependence plots for (c) rs1064173 (*HLA-DQB1*) and (d) rs689 (*INS*). Values between an allele dosage of 0 and 1, or 1 and 2 were interpolated for visualization purposes.

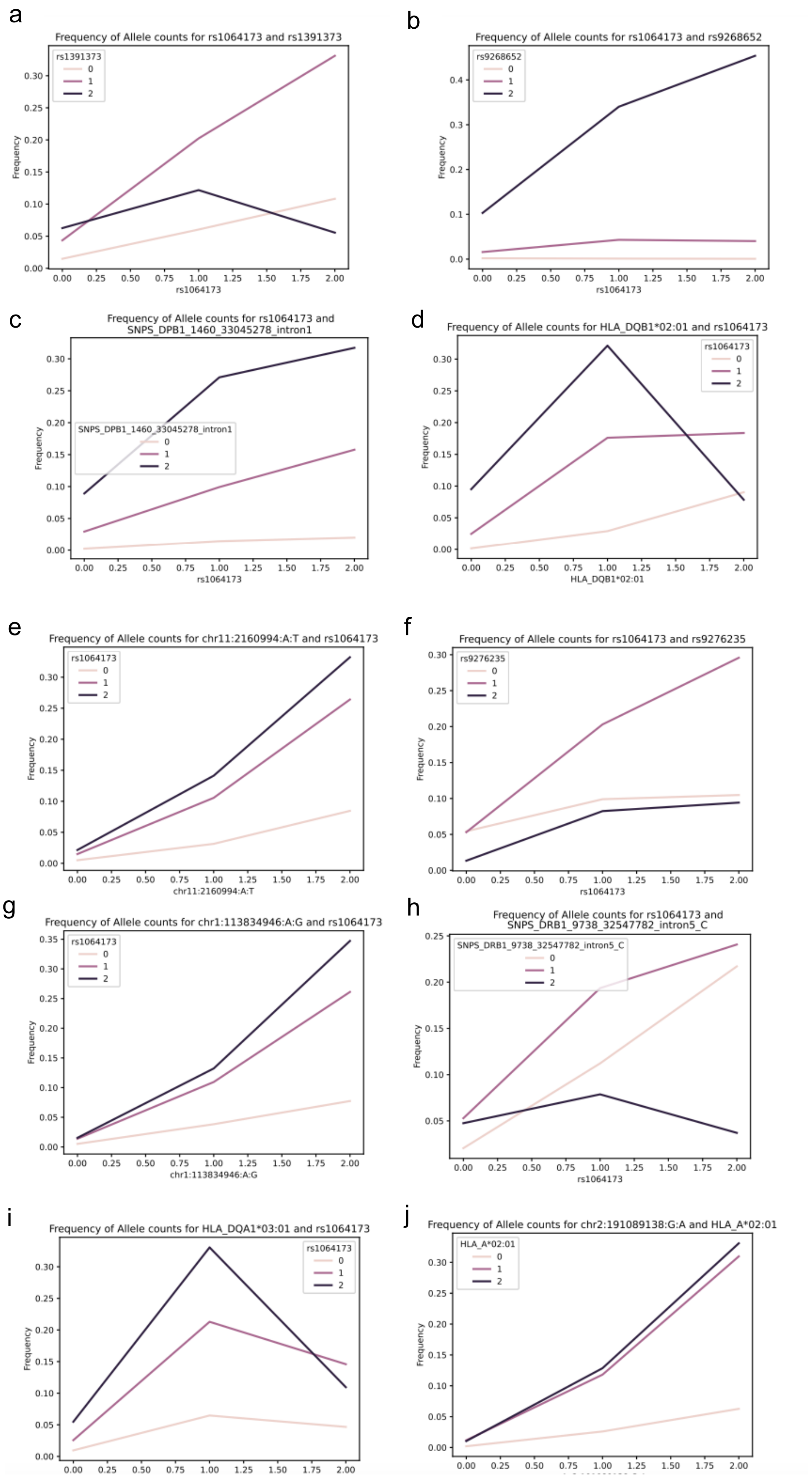

a
Frequency of Allele counts for rs1064173 and rs1391373
rs1391373
0
1
2
Frequency
rs1064173
b
Frequency of Allele counts for rs1064173 and rs9268652
rs9268652
0
1
2
Frequency
rs1064173
c
Frequency of Allele counts for rs1064173 and SNPS_DPB1_1460_33045278_intron1
SNPS_DPB1_1460_33045278_intron1
0
1
2
Frequency
rs1064173
d
Frequency of Allele counts for HLA_DQB1*02:01 and rs1064173
rs1064173
0
1
2
Frequency
HLA_DQB1*02:01
e
Frequency of Allele counts for chr11:2160994:A:T and rs1064173
rs1064173
0
1
2
Frequency
chr11:2160994:A:T
f
Frequency of Allele counts for rs1064173 and rs9276235
rs9276235
0
1
2
Frequency
rs1064173
g
Frequency of Allele counts for chr1:113834946:A:G and rs1064173
rs1064173
0
1
2
Frequency
chr1:113834946:A:G
h
Frequency of Allele counts for rs1064173 and SNPS_DRB1_9738_32547782_intron5_C
SNPS_DRB1_9738_32547782_intron5_C
0
1
2
Frequency
rs1064173
i
Frequency of Allele counts for HLA_DQA1*03:01 and rs1064173
rs1064173
0
1
2
Frequency
j
Frequency of Allele counts for chr2:191089138:G:A and HLA_A*02:01
HLA_A*02:01
0
1
2
Frequency

Supplementary Figure 6. Allele Frequencies between Interacting Variants. Frequency of allele counts for the top ten pairs of interacting variants ranked by interaction value z-score (see Supplementary Table 13 for details). Points were graphed for allele dosages of 0, 1 and 2 of the risk-associated allele or haplotype. Lines were drawn through plotted points for visualization purposes.

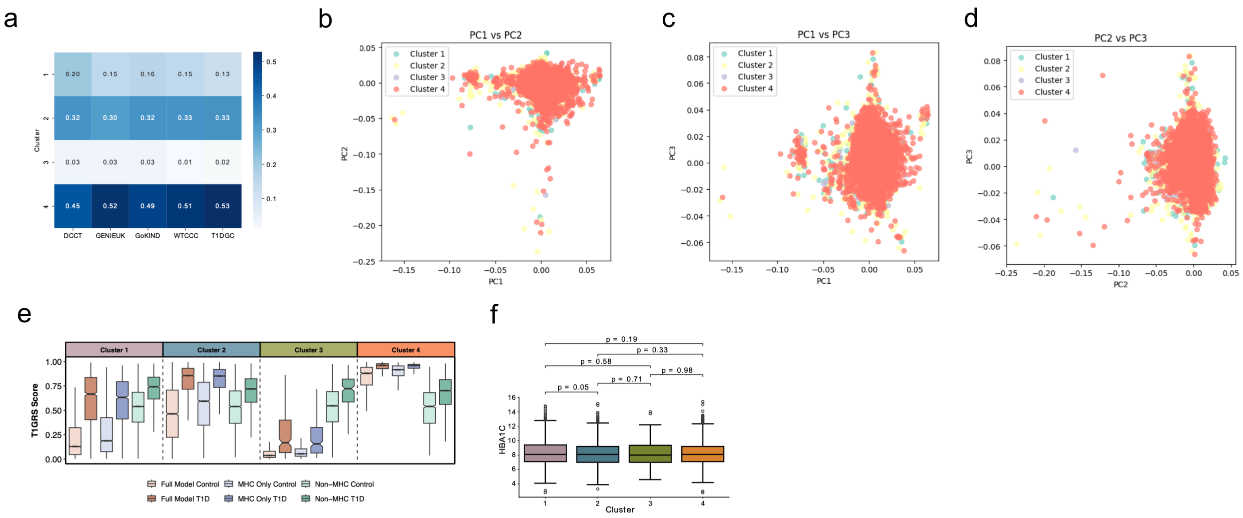

Supplementary Figure 7. T1GRS Clusters Demonstrate No Enrichment of Study Cohort, Population Sub-structure, T1GRS Scores, or HbA1C level. (a) Heat map with the fraction of individuals per cluster (T1GRS discovery cohort) in each cohort. (b-d) Individuals in T1GRS discovery cohort projected along the first three principal components generated using all imputed genotypes with MAF > 1%. Points are colored by T1GRS cluster assignment. (e) Per-cluster break down of T1GRS-var scores for full model, MHC-only model, and non-MHC model. Box-and-whisker plots show median value and interquartile range. (f) HBA1c measurements broken down by cluster. P values were calculated using two-sample, two-tailed t-tests comparing each pair of clusters. Box-and-whisker plots show median value and interquartile range.
