# Supplementary material for: Genetic association and machine learning improve the prediction of type 1 diabetes risk
Source: Nat Genet. 2026 Apr 30;58(5):1062–72. doi: 10.1038/s41588-026-02578-y (PMC13175890; doi:10.1038/s41588-026-02578-y)
Supplement: Supplementary file 2 — Reporting Summary [file 41588_2026_2578_MOESM2_ESM.pdf]

nature portfolio

Corresponding author(s): Gaulton, McGrail

Last updated by author(s): Mar 8, 2026

Reporting Summary

Nature Portfolio wishes to improve the reproducibility of the work that we publish. This form provides structure for consistency and transparency in reporting. For further information on Nature Portfolio policies, see our Editorial Policies and the Editorial Policy Checklist.

Statistics

For all statistical analyses, confirm that the following items are present in the figure legend, table legend, main text, or Methods section.

n/a	Confirmed	
☐	☒	The exact sample size (*n*) for each experimental group/condition, given as a discrete number and unit of measurement
☐	☒	A statement on whether measurements were taken from distinct samples or whether the same sample was measured repeatedly
☐	☒	The statistical test(s) used AND whether they are one- or two-sided<br>*Only common tests should be described solely by name; describe more complex techniques in the Methods section.*
☐	☒	A description of all covariates tested
☐	☒	A description of any assumptions or corrections, such as tests of normality and adjustment for multiple comparisons
☐	☒	A full description of the statistical parameters including central tendency (e.g. means) or other basic estimates (e.g. regression coefficient) AND variation (e.g. standard deviation) or associated estimates of uncertainty (e.g. confidence intervals)
☐	☒	For null hypothesis testing, the test statistic (e.g. *F*, *t*, *r*) with confidence intervals, effect sizes, degrees of freedom and *P* value noted<br>*Give P values as exact values whenever suitable.*
☐	☒	For Bayesian analysis, information on the choice of priors and Markov chain Monte Carlo settings
☐	☒	For hierarchical and complex designs, identification of the appropriate level for tests and full reporting of outcomes
☒	☐	Estimates of effect sizes (e.g. Cohen's *d*, Pearson's *r*), indicating how they were calculated

Our web collection on statistics for biologists contains articles on many of the points above.

Software and code

Policy information about availability of computer code

Data collection	No code was used to collect data, pre-existing sample genotypes were obtained from dbGAP and other controlled-access repositories and biobanks.
Data analysis	For preparation of sample genotypes, we used the HRC imputation preparation program (version 4.2.9, https://www.well.ox.ac.uk/~wrayner/tools/) and PLINK 1.9 to QC filter variants and samples. We used TOPMed and Michigan HLA imputation servers to impute genotypes into TOPMED r2 panel and Michigan HLA four-digit Multi-ethnic HLA reference panel (v1). We used EPACTS v3.3.0 to perform association analyses. We used SUSIE v0.11.42 to perform fine-mapping. We used fimo v4.12.0 to annotate variants at novel signals. CatBoost v1.0.6 was used to train the machine learning model. We used SHAP v0.41.0 to interpret features from the machine learning model. We used Scanpy v1.8.2 to perform sample clustering.<br><br>Custom code used to create the T1GRS model is provided at doi:10.5281/zenodo.18842258 and https://github.com/Gaulton-Lab/t1d-grs-analysis-catboost.

For manuscripts utilizing custom algorithms or software that are central to the research but not yet described in published literature, software must be made available to editors and reviewers. We strongly encourage code deposition in a community repository (e.g. GitHub). See the Nature Portfolio guidelines for submitting code & software for further information.

Data

Policy information about availability of data

All manuscripts must include a data availability statement. This statement should provide the following information, where applicable:

- Accession codes, unique identifiers, or web links for publicly available datasets
- A description of any restrictions on data availability
- For clinical datasets or third party data, please ensure that the statement adheres to our policy

Summary statistics from the T1D GWAS are available in the GWAS Catalogue under accession number GCST90824163.

Research involving human participants, their data, or biological material

Policy information about studies with human participants or human data. See also policy information about sex, gender (identity/presentation), and sexual orientation and race, ethnicity and racism.

Reporting on sex and gender	Both sexes were included in the analysis of this study.
Reporting on race, ethnicity, or other socially relevant groupings	We performed the main analyses using individuals of European ancestry defined by cohort criteria and PC analysis. We also tested the model using African Ancestry cohorts (CLEAR and SEARCH) to provide insight into how a European ancestry risk model can be applied to individuals of other ancestries.
Population characteristics	Samples were used based on T1D diagnosis and individuals without diabetes were selected based on matched ancestry and genotype array where possible. Genotype data was used to generate PCs to control for ancestry level population structural differences in the European ancestry samples.
Recruitment	Sample data were obtained from pre-existing cohorts for individuals with and without T1D
Ethics oversight	The IRB of the University of California San Diego approved the use of human genetic data.

Note that full information on the approval of the study protocol must also be provided in the manuscript.

Field-specific reporting

Please select the one below that is the best fit for your research. If you are not sure, read the appropriate sections before making your selection.

☒ Life sciences ☐ Behavioural & social sciences ☐ Ecological, evolutionary & environmental sciences

For a reference copy of the document with all sections, see nature.com/documents/nr-reporting-summary-flat.pdf

Life sciences study design

All studies must disclose on these points even when the disclosure is negative.

Sample size	817,718 samples (20,355 T1D, 797,363 non-diabetes) of European ancestry were used for genetic association analyses where the sample size was based on those available in cohorts with T1D case and control individuals from controlled-access repositories and biobanks. The sample size represents the largest genetic association study of T1D. No methods were used to predetermine sample size.
Data exclusions	Samples were excluded during data preparation if samples did not pass pre-established quality control metrics including (i) missing genotypes (missing >5%), (ii) sex mismatch with phenotype records (homchrX > 0.2 for females and homchrX < 0.8 for males), (iii) cryptic relatedness through identity-by-descent (IBD >0.2), and (iv) non-European ancestry through PCA with 1000 Genomes Project30 (>3 interquartile range from 25th and 75th percentiles of European 1KGP samples on the first four PCs).
Replication	Novel T1D loci were replicated using a larger T1D cohort in Finngen. Five loci did not reach the genome-wide significance threshold for the study and therefore were considered putative loci that still require further validation. For the machine learning model, independent cohorts (2,439 T1D, 79,601 non-diabetes) not used in training were used to validate model performance. For the genetic clustering analyses, cluster membership and phenotypic associations were validated in independent cohorts not used to identify the clusters.
Randomization	Individuals were assigned to groups based on T1D status defined from cohort information, and therefore randomization is not applicable.
Blinding	Blinding of data generation was not applicable to this study as the genotyping data and calls were pre-existing. Processing, imputation and filtering of sample genotypes are performed agnostic to disease status.

Reporting for specific materials, systems and methods

We require information from authors about some types of materials, experimental systems and methods used in many studies. Here, indicate whether each material, system or method listed is relevant to your study. If you are not sure if a list item applies to your research, read the appropriate section before selecting a response.

Materials & experimental systems

n/a	Involved in the study
☒	☐ Antibodies
☒	☐ Eukaryotic cell lines
☒	☐ Palaeontology and archaeology
☒	☐ Animals and other organisms
☒	☐ Clinical data
☒	☐ Dual use research of concern
☒	☐ Plants

Methods

n/a	Involved in the study
☒	☐ ChIP-seq
☒	☐ Flow cytometry
☒	☐ MRI-based neuroimaging

Plants

Seed stocks	N/A
Novel plant genotypes	N/A
Authentication	N/A
